# Resilience patterns of human mobility in response to extreme urban floods

**DOI:** 10.1093/nsr/nwad097

**Published:** 2023-04-11

**Authors:** Junqing Tang, Pengjun Zhao, Zhaoya Gong, Hongbo Zhao, Fengjue Huang, Jiaying Li, Zhihe Chen, Ling Yu, Jun Chen

**Affiliations:** School of Urban Planning and Design, Shenzhen Graduate School, Peking University, Shenzhen 518055, China; Key Laboratory of Earth Surface System and Human-Earth Relations of Ministry of Natural Resources of China, Shenzhen Graduate School, Peking University, Shenzhen 518055, China; School of Urban Planning and Design, Shenzhen Graduate School, Peking University, Shenzhen 518055, China; Key Laboratory of Earth Surface System and Human-Earth Relations of Ministry of Natural Resources of China, Shenzhen Graduate School, Peking University, Shenzhen 518055, China; School of Urban and Environmental Sciences, Peking University, Beijing 100871, China; School of Urban Planning and Design, Shenzhen Graduate School, Peking University, Shenzhen 518055, China; Key Laboratory of Earth Surface System and Human-Earth Relations of Ministry of Natural Resources of China, Shenzhen Graduate School, Peking University, Shenzhen 518055, China; Key Research Institute of Yellow River Civilization and Sustainable Development & Collaborative Innovation Center on Yellow River Civilization, Henan Province and Ministry of Education, Henan University, Kaifeng 475001, China; School of Urban Planning and Design, Shenzhen Graduate School, Peking University, Shenzhen 518055, China; School of Urban Planning and Design, Shenzhen Graduate School, Peking University, Shenzhen 518055, China; School of Urban Planning and Design, Shenzhen Graduate School, Peking University, Shenzhen 518055, China; School of Urban Planning and Design, Shenzhen Graduate School, Peking University, Shenzhen 518055, China; Key National Geomatics Center of China, Beijing 100830, China

**Keywords:** disaster resilience, urban flood, human mobility, Zhengzhou flood, mobile phone data

## Abstract

Large-scale disasters can disproportionately impact different population groups, causing prominent disparity and inequality, especially for the vulnerable and marginalized. Here, we investigate the resilience of human mobility under the disturbance of the unprecedented ‘720’ Zhengzhou flood in China in 2021 using records of 1.32 billion mobile phone signaling generated by 4.35 million people. We find that although pluvial floods can trigger mobility reductions, the overall structural dynamics of mobility networks remain relatively stable. We also find that the low levels of mobility resilience in female, adolescent and older adult groups are mainly due to their insufficient capabilities to maintain business-as-usual travel frequency during the flood. Most importantly, we reveal three types of counter-intuitive, yet widely existing, resilience patterns of human mobility (namely, ‘reverse bathtub’, ‘ever-increasing’ and ‘ever-decreasing’ patterns), and demonstrate a universal mechanism of disaster-avoidance response by further corroborating that those abnormal resilience patterns are not associated with people’s gender or age. In view of the common association between travel behaviors and travelers’ socio-demographic characteristics, our findings provide a caveat for scholars when disclosing disparities in human travel behaviors during flood-induced emergencies.

## Teaser

From China's most severe and unexpected urban flood event in recent years, we unravel the heterogeneity of resilience patterns in large-scale human mobility using a massive amount of mobile phone signaling data. For both scholars and practitioners, this paper provides insights to further understand the complex behaviors in collective human movements against severe climate-induced urban floods and sheds new light on the evidence-informed decision-makings for socially vulnerable populations when building urban resilience.

## INTRODUCTION

As a major driver of mounting natural disasters worldwide, climate change is among the most intractable challenges we are facing in the twenty-first century [1,2]. According to the United Nations Office for Disaster Risk Reduction, climate- and weather-related calamities have surged five-fold over the past 50 years [3]. Because of the fast pace of urbanization and infrastructure aging, the climate vulnerability of communities, cities and metropolitan regions has significantly amplified [4]. Floods have been one of the most devastating natural hazards, causing the highest insured losses in cities around the world [5]. The latest report issued by the Swiss Re Institute revealed that floods accounted for 31% of total global losses from natural catastrophes (a total of US $82 billion in 2021) [6]. A recent study estimated that about 1.81 billion people (23% of the world’s population) are directly exposed to a significant level of flood risks; developing countries in Asia comprise the majority (e.g. 395 million in China and 390 million in India, accounting for over one-third of global exposure) [7].

From its origin as a scientific term in early nineteenth-century materials science, the concept of *resilience* has been popular in multiple fields [8]. Furthermore, its application has broadened to embrace a more multidimensional interpretation of its core. In flood-risk research, the concept mainly describes the ability to successfully resist flood impacts and quickly restore basic performance to mitigate losses [9]. Scholars have also reached a common agreement on using the classic paradigm of *bathtub-shaped resilience curves* to depict drawdown-drawup systemic responses in the before–during–after process against floods [10]. This paradigm is so fundamental and essential that it has become the cornerstone of resilience-allied studies [11].

From the standpoint of human movement behaviors in cities, human mobility has been extensively studied for decades, not least because it provides useful insights into how we interact with each other and move from place to place [12,13], but as much because it can fundamentally answer questions concerning the governing laws of dynamic growth and evolution of our cities [14–17] and shed insightful lights on epidemic research in recent years [18–21]. Human mobility forms a highly complex and dynamic urban system that is variable and sensitive to external disruptions  [22–24]; it is, thus, intriguing to see how complex human movement activities react to the impacts of urban disasters.

It is already well documented that population groups with diverse demographic and socioeconomic characteristics behave differently and are associated with pervasive disparities and inequalities [25–28]. Scholars have shown that such inequality can be even worse and more pronounced in socially vulnerable groups [29]. For instance, a recent study using data on 90 million individuals’ mobility behavior in the United States revealed significant disparities in mobility changes among population groups in the case of COVID-19, where residents from low-income neighborhoods were forced to return to their pre-pandemic commuting behaviors faster than those from high-income communities who could afford a longer stay-at-home period [30]. Also, the risks of various types of disasters are pervasively high for older adults and females, who are more prone to a greater rate of sickness, economic loss, family burden and post-traumatic stress [31–34], which consequently leads to a disproportionately higher vulnerability and lower resilience among these populations [35]. These disparities and inequalities are also revealed in flood-specific studies [26,36], covering both short-term daily mobility and long-term migration mobility in the face of different sorts of flood disasters [37], including sea-level-rise-related inundation hazards [38,39].

Acknowledging these pervasive disparities and inequalities in different populations is important, but this perpetuated perception can also sometimes lead to significant limitations when investigating mobility resilience for more efficient risk-mitigation measures. First, with this prior knowledge of travel behavior disparities, earlier studies merely revealed that the overall changes in either holistic or group-level human mobility follow conventional ‘*bathtub-shaped resilience curves*’ with different parabolic shapes [8,13,30], which overlooks further explorations of the heterogeneous mobility resilience of possibly more diverse patterns in space. Second, this perception can inhibit researchers from probing possible common mechanisms of disaster-induced behavior responses that lie beneath surface-level disparities [36]. It is, therefore, necessary to look beneath such disparities and better understand the resilience patterns of how people collectively move in response to urban floods for a more sustainable and equitable long-term development [8,40]. Nonetheless, such understandings still remain under-researched, especially for vulnerable groups in developing countries, such as China [7,41].

**Figure 1.**
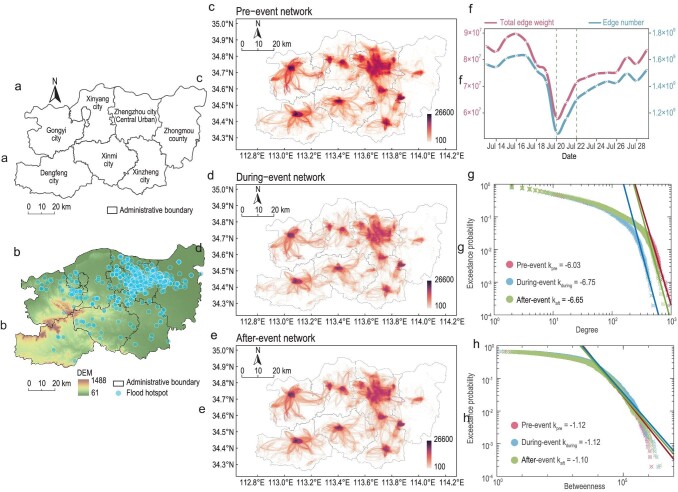
The study area and spatial-temporal evolution of the mobility network *G* with flow intensity *W* > 100 (i.e. the edge weight—flux on the edges of greater than 100 trips). Note that the daily average origin-destination (OD) flux at each disaster stage was first obtained before filtering the edge weights with the intensity threshold. (a) Administrative boundary of the Zhengzhou region. (b) Elevation (DEM) of the Zhenzhou region and the geolocation of the flood damage hotspots. These hotspots are labeled as 4-km buffer zones for visualization, denoting places where intense rescue activities occurred based on the recordings from Weibo (the largest social media platform in China), which also show the proxy locations of the most severe damage in infrastructure systems during the event. (c–e) Visualization of the daily average *G*_*W* > 100_ network before, during and after the flood event, respectively. (f) Changes in the networks’ total edge weights and edge numbers within the observation window. (g and h) Exceedance probability distributions of degree and betweenness of the networks. Note the clear gap in the tails between the during-event distribution and the other two stages in (g). See Section 2.1 within the online supplementary material for similar observations in *G*_50 <*W*<100_ and *G*_*W*<50_ networks.

Hence, in what follows, we address these limitations by investigating the resilience patterns of human mobility in detail, using the ‘720’ Zhengzhou flood event, in China, as a case study. First, we quantify the resilience level and disclose the disparities in mobility resilience in demographic groups using dynamic network simulation models; next, we investigate the resilience patterns of human mobility and its association with travelers’ socio-demographic attributes using discrete choice models. We collect high-fidelity, large-volume mobile phone signaling data—1.32 billion records generated by 4.35 million travelers (comprising 34.3% of the total population in the region)—and other multisource geolocation and socioeconomic data for the investigation. This event was specifically chosen as it was one of the most severe urban pluvial floods to have occurred around the globe in recent years and also the most powerful one with a magnitude not previously experienced since Henan province began publicly reporting rainfall disasters. On July 20, 2021, a record-breaking heavy rainfall occurred in the Zhengzhou region, Henan province, China, an inland densely populated region with a total area of 7600 km^2^, 12.7 million residents and a 79.1% urbanization rate (see Fig. 1a and b and Sections 1.1 and 1.2 within the online supplementary material). The region is located in a semiarid area and is known for its relatively low average annual precipitation [42]. This unexpected heavy rainfall led to an extreme urban flood, causing massive injuries to the public and losses in critical infrastructure systems, with a death toll of 380; nearly two million people were affected [43]. Very quickly, this catastrophic event in central China drew extensive attention globally [44,45]. Owing to the growing availability of mobile phone big data, we can now uncover more details regarding how people collectively moved and responded to this unprecedented urban flood disaster.

## RESULTS

### Movement patterns and mobility resilience

To begin with, we first look at the overall movement patterns during different stages of the disaster over the entire study area. Based on the open news of the flood and hourly precipitation reports, we reconstructed the entire storyline and selected 17 consecutive days as the observation window for the analysis and divided them into three stages (see Figs S2–S3 in Section 1.2 of the online supplementary material). Because the most intensive rainfall occurred on July 20 and continued, with a decreasing intensity, from July 21 to 22, this three-day window is defined as the ‘during-event’ stage. The period seven days before the event (i.e. July 13 to 19) is the pre-event stage and that seven days after the event (i.e. July 23 to 29) is referred to as the post-event stage.

By discretizing the entire study area into 1 × 1 km^2^ grids (see the Methods section for details), we constructed mobility networks with the origin and destination of spatial-temporal movements as nodes and the trips as edges. Panels c–e of Fig. 1 show the geographical changes in the mobility networks at different disaster stages. Visually, one can observe that the network indeed becomes less dense during the flood, and this is confirmed by the reduced edge number and total edge weight (i.e. the total flow intensity in the network) in Fig. 1f, indicating that overall human mobility affected by the disaster has significantly dropped. For the network nodes (i.e. the OD locations), while the probability distribution of the betweenness centrality (i.e. those critical bridges in the network) remained relatively unvaried, the distribution of degree (i.e. network connectivity) has been clearly affected during the event, where those extremely large degrees were diminished. Together, this indicates that the flood disaster not only weakened the overall mobility intensity, but also reduced the number of those very large hubs in the during-event stage (Fig. 1g). However, an adequately stable fat-tail effect can still be identified across all three stages (i.e. the power-law distribution)—panels g and h of Fig. 1 show that the *k* values did not drastically change in different disaster stages, suggesting a relatively unchanged non-trivial scale-free dynamics of network structures.

**Figure 2.**
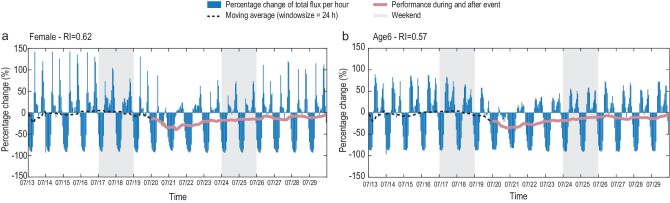
Time-series performance of population groups and the quantified resilience scores: (a) female group and (b) age group 6 (i.e. those over 60 years). The total flux defined here is the total edge weight of a network at each time step (i.e. an hourly basis). The black dotted and red lines are moving averaged values of the normalized percentage changes of mobility performance based on the pre-event benchmarks. See Section 2.2 within the online supplementary material for detailed descriptions regarding how the performance profiles and resilience scores were prepared and calculated, respectively.

Given the reduction in overall mobility, we turned our attention to how resilient the different demographic groups were in response to this flood, with a focus on two particular characteristics: gender (males and females) and age groups (1, 0–18; 2, 19–29; 3, 30–39; 4, 40–49; 5, 50–59; and 6, over 60 years old). By applying the resilience quantification method and the statistical tests (see the Methods section for details), we obtained the quantified scores of mobility resilience based on the percentage change in daily averaged mobility (Fig. 2). The resilience calculation for males and females yields scores of 0.71 and 0.62, respectively, indicating that male travelers have more resilient mobility than females (Tukey’s test *p* ≪ 0.001 under the 95% confidence interval, suggesting a statistically significant difference). The results obtained for the six age groups are 0.52, 0.67, 0.67, 0.64, 0.62 and 0.57, respectively, which implies that travelers aged 19–29 and 30–39 relatively have the same highest level of mobility resilience in the population, whereas those younger than 18 and those older than 60 are both least resilient (see Section 2.3 within the online supplementary material for statistical tests on the significance). The results verify the intuitive disparities of mobility resilience across different groups, wherein vulnerable groups are more likely to be impacted by the flood event and therefore suffer from a lower mobility resilience level.

### Modeling the disparities of mobility resilience levels

Based on the observed power-law features, we devised a preferential attachment model, the dual Barabàsi–Albert (DBA) model [46], to model the dynamic growth and shrinkage process of mobility networks, and to quantitatively explain the observed disparity in the mobility resilience across demographic groups. The model has three critical parameters, *m*_1_, *m*_2_ and *p*, where *p* is the probability of generating *m*_1_ edges that link the existing nodes in the network based on the preferential attachment principle (see the Methods section for details). A larger *p* indicates that a network has a higher probability of actively generating *m*_1_ new origin-destination (OD) connections in each time step and a lower probability of 1 − *p* of generating only one new connection each time (see Fig. S8 in Section 2.3 of the online supplementary material).

**Figure 3.**
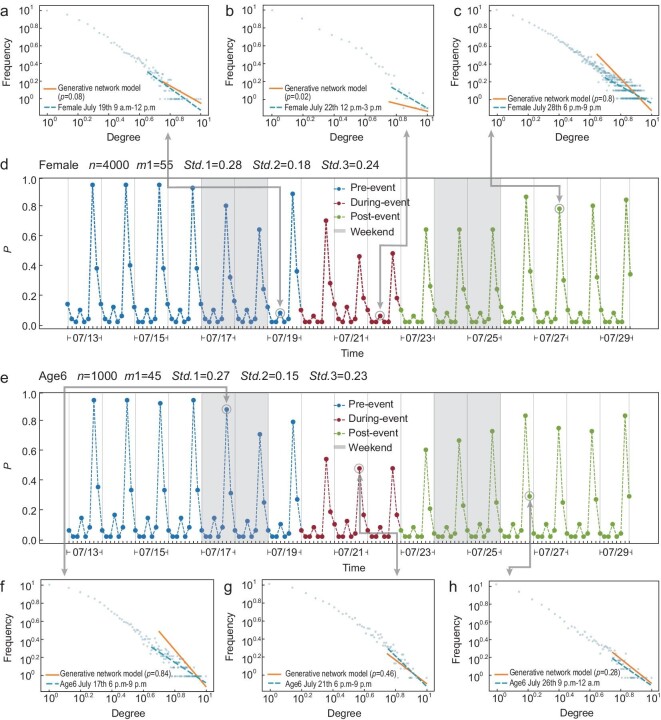
DBA model fittings for the two selected vulnerable groups. The entire evolutionary process of the overall mobility network was first divided on an hourly basis into slices and then we averaged every sliced network into 3-h time slots. Panels (a–c) and (f–h) show the goodness of the power-law fitting between ground-truth networks and the DBA-generated networks in randomly selected slices. Panels (d) and (e) are generation probability *p* values for model fitting in the female group and older adult group, respectively. Note that *n* denotes the number of nodes in the generated network, *m*_1_ is the number of edges to link the new node to existing nodes with probability *p*, and Std.1, Std.2 and Std.3 represent standard deviations at the pre-, during and post-event stages, respectively, indicating the volatility of the time series

The model adequately captures the dynamic changes in the mobility networks, as we can see an obvious drop in *p* during the disaster for all groups (Fig. 3). The power-law feature of the mobility networks is also well captured by the model. The changing trend of standard deviations across the three stages confirms the volatility of the changes of probability *p*; this indicates that the probability of generating more connections by taking a larger amount of trips during the event significantly declines in all groups (as for the daily average, the probability drops from $\overline{p}_{\rm before}=0.21$ to $\overline{p}_{\rm during}=0.14$ for all groups; the average daily maximum probability falls from $\overline{p}_{\rm before}^{\rm max}=0.89$ to $\overline{p}_{\rm during}^{\rm max}=0.56$ for all groups). From the modeling results, we also found that young, older and female travelers tend to have much smaller values of *m*_1_ compared to other groups ($m_{1}^{\rm Age,group1}=10$, $m_{1}^{\rm Age,group6}=45$ and $m_{1}^{\rm female}=55$, respectively) (see Table S1 in Section 2.3 of the online supplementary material). Essentially, a small *m*_1_ value and a low *p* level in the during-event stage explain the less dense networks of movement activities. In addition, combined with their relatively more volatile downturns of the *p* level, the outcomes quantitatively illustrate that the probability of maintaining the business-as-usual (i.e. pre-disaster) travel frequency in these vulnerable groups is much lower than other groups during the event, which explains the lower resilience level and insufficient post-event recovery we revealed in the previous section.

### Diverse resilience patterns in response to the disaster

The paradigm of resilience pattern is the classic *‘bathtub-shaped resilience curve’* (or the ‘drawdown-drawup’ curve) [10,11]; this is also widely known as the resilience-triangle model (see Fig. S7 in Section 2.2 of the online supplementary material). In this paradigm, intuitively, a time-series performance level decreases when disasters strike (i.e. the drawdown: the difference between the pre-event and the during-event levels should be a positive value as the former is higher than the latter). In the recovery process, the performance curve gradually rises due to restoration (i.e. the drawup: the difference between the during-event and the post-event levels should be a negative value as the former is at a lower level than the latter). This ‘drawdown-and-drawup’ perspective is the cornerstone of resilience literature.

**Figure 4.**
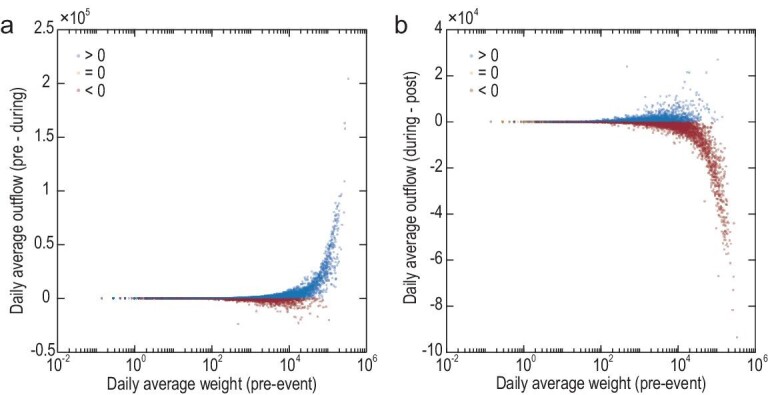
Outliers of mobility patterns in both the drawdown and drawup processes in the spatial grids. (a) Outlier identification in the drawdown process (i.e. the pre-event level minus the during-event level). (b) Outlier identification in the drawup process (i.e. the post-event level minus the during-event level). This plot is based on the observation of outflow patterns; similar characteristics can be found in the inflow patterns. The blue dots indicate that the differences between the two compared levels are positive, while the red dots represent negative values. To interpret, for example, in the drawdown process, the conventional performance of the difference between the pre- and during-event levels should be a positive number (i.e. a blue dot), whereas it is clear that a large number of red dots exist. A similar counter-intuitive performance can also be observed in the drawup process.

However, by analyzing the ‘drawdown-drawup’ performance of daily averaged mobility flows in all spatial units, we found a significant number of abnormal patterns at the drawdown and drawup processes from both outflow and inflow cases (Fig. 4). In other words, there is a significant number of places having a drawup pattern when they should drawdown and vice versa. For the abnormal points in the drawdown process, it indicates that mobility was counter-intuitively stimulated by the disaster and increased, and those abnormalities in the drawup process imply that mobility surprisingly declines during the post-event recovery. Additionally, we can observe that those abnormal outliers are more likely to concentrate in the grids with a higher pre-event daily averaged flux. Considering outflows and inflows, this implies that the stronger the daily averaged flow intensity at a place before the disaster (i.e. the more travelers depart from this place as their origin or arrive at this place as their destination), the higher the likelihood of the place having abnormal mobility resilience patterns when a disaster strikes.

**Figure 5.**
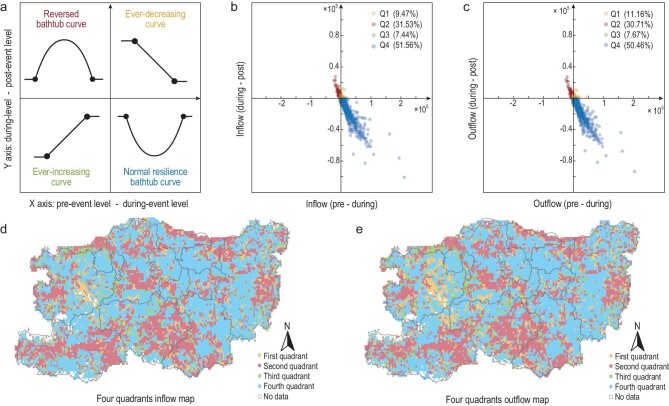
Distributions of the four types of resilience patterns. (a) An illustration of the heterogeneous types of resilience patterns found in the study area. (b and c) Quadrant distributions of the resilience patterns concerning the inflow and outflow of the OD movements. (d and e) Spatial distributions of the resilience patterns concerning the inflow and outflow of the OD movements. The color code for labeling the four quadrants is consistent in all subplots.

We then mapped the mobility patterns onto the four quadrants, with the drawdown and drawup processes as the two axes, and revealed four dissimilar types of mobility resilience patterns (Fig. 5). Among these abnormalities, the three types of unusual resilience patterns are the ‘reversed bathtub curve’, the ‘ever-decreasing curve’ and the ‘ever-increasing curve’. In terms of spatial distribution, there is an unexpectedly high occupation of abnormal resilience patterns, which account for up to half of the total number of spatial units (i.e. on average, the ratio between normal and abnormal mobility resilience patterns is approximately 1:1). This ratio was tested at a smaller spatial scale, and its relative scale invariance was also verified (i.e. the sub-network community level; see Section 3.2 within the online supplementary material). Most importantly, we found that, with only a minor difference between inflows and outflows in spatial distribution, the normal resilience pattern (blue spots) seems to distribute uniformly in the study area due to the large scale of the flood, while the reversed bathtub resilience pattern (red spots) is more concentrated in the southern region, where those unaffected areas are located. The rest of the resilience patterns (i.e. ever-increasing and ever-decreasing curves) tend to distribute on the peripheries of the red spots, forming narrow corridors and small agglomerations in the western and eastern regions.

Taking the inflow as an example, the reversed bathtub pattern (accounts for approximately 30%) confirms that this flood disaster stimulates inflow mobility instead of suppressing it in those places. Meanwhile, in the post-event recovery process, the high mobility inflow dissipates to a lower level. This could reflect disaster-avoidance behaviors in which people temporarily alter their travel route choices to agglomerate at certain new spots during the flood, but after the event, they shift back to their regular routes. The ever-increasing pattern (approximately 10%) indicates that the disaster stimulates inflow during the flood, causing a swift agglomeration of arriving travelers. This trend is maintained, even after the event, in the drawup recovery stage. One possible explanation for this could be that the travelers form and maintain new travel routines after the event, for example, a new route for commuting, an intentional detour to avoid certain places for safety or an unwilling detour due to roads being blocked by long-standing water after the flood. On the contrary, the ever-decreasing curve (also accounting for approximately 10%) implies that the disaster suppresses inflow in this area, and this decreasing tendency continues after the disaster. This suggests that the disaster may have harmed the spatial attractiveness of those places for a longer period, causing travelers to avoid those spots even after the event due to safety concerns and inaccessible routes.

### Abnormal resilience patterns explained by gender and age

Moving forward, we explored whether this heterogeneity of resilience patterns could be explained by the travelers’ demographic attributes. In other words, we would like to see if the abnormal resilience patterns are associated with the vulnerable groups due to their less resilient mobility. This part of the analysis consists of two steps. First, we checked whether we needed to consider the spatial effect in causal inference (i.e. would there be spillover effects from neighboring grids to the target grid?). We conducted a bivariate global Moran’s I test for each quadrant type with respect to demographic traits. Second, based on the findings from the first step, we established a series of penalized multinomial logistic (MNL) regression models to check the explainable strength of demographical characteristics in terms of modeling quadrant types. To eliminate the influence of variations in the total population in different grids, we converted the mobility flow values under all demographical groups into relative ratios.

Bivariate Moran’s I examines the spatial correlations between resilience patterns (i.e. quadrant types) and all gender and age groups (i.e. demographic attributes). We applied the Euclidean distance and set the distance threshold to 1719.68 m based on the distance decay, resulting in a mean number of 13.51 neighbors for each grid. As shown in Table 1, we found that all demographic groups have significantly small correlation coefficients in the four quadrants, which indicates statistically insignificant relationships in this bivariate correlation analysis (with *p* ≫ 0.01). The results strongly suggest that there is no obvious agglomeration or diffusion phenomenon of the demographic attributes with respect to different mobility resilience patterns in space, that is, the spatial relationships, or the spillover effects, from the neighboring grids can be neglected when constructing the regression models in the next step.

**Table 1.** Global bivariate Moran’s I between the four quadrants and all demographic groups in terms of inflow.

			Age	Age	Age	Age	Age	Age
	Female	Male	group 1	group 2	group 3	group 4	group 5	group 6
Q1	−0.015	0.015	−0.003	−0.013	−0.019	0.001	0.022	0.014
Q2	0.013	−0.013	0.017	−0.043	−0.004	0.014	0.021	0.012
Q3	−0.005	0.005	−0.006	−0.049	−0.021	0.003	0.038	0.040
Q4	0.000	0.000	0.010	0.073	0.026	−0.015	−0.052	−0.040

*Note.* Because the difference between inflow and outflow is minor, we only present inflow hereafter.

**Figure 6.**
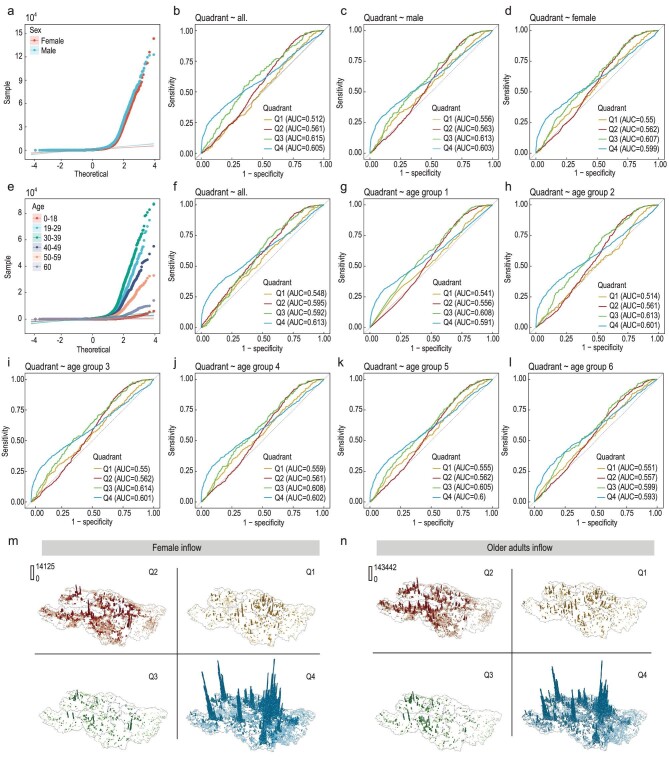
Receiver operating characteristic (ROC) curves for the developed penalized MNL models and the spatial distributions of female and older adult travelers’ daily averaged inflows against the four different quadrants of the resilience patterns. (a) Results of the normality test for all gender variables. (b) ROC curves and AUC values of the model fitting with both gender variables. (c,d) ROC curves and AUC values of the model fitting with males and females, respectively. (e) Results of the normality test for all age variables. (f) ROC curves and AUC values of the model fitting with all age variables. (g–l) ROC curves and AUC values of the model fitting with each age variable, respectively. (m and n) Spatial distributions of female and older adult travelers’ daily average inflows against the four different quadrants, respectively. Note that, for comparative illustration, female inflow × 0.5 and older adult inflow × 5. Similar observations are made when analyzing the other groups (see the analysis of the male group as an example in Section 3.4 within the online supplementary material).

Figure 6 visualizes the spatial distributions of the four quadrants in the female group and age group 6 (see Fig. S14 in the online supplementary material for comparison with the male group), and shows the explainable strength of the demographic traits, the explainable variables, for modeling the multiclass quadrants, the responsive variables. In addition, we also performed a stepwise regression analysis to determine the detailed explainable strength of each individual explainable variable (see Tables S4– S7 in Section 3.3 of the online supplementary material). The high variance inflation factor (VIF) and low area under curve (AUC) values denote strong multicollinearity with unacceptable model predictability (for the stepwise regressions with gender, VIF_female_ = 11 288.1, VIF_male_ = 7204.0, AUC_average_ = 0.58, and averaged model accuracy is 34%; for the stepwise regressions with age, VIF_average_ = 999 486.2, VIF_min_ = 248.4, AUC_average_ = 0.58 and averaged model accuracy is 30%; see Section 3.3 within the online supplementary material for more details on the test results). Similar findings can also be confirmed in the outflow case. The analytical results signal that both normal and abnormal mobility resilience patterns are not essentially associated with either gender or age. In other words, this shows that on a collective scale whether the dynamic human movements in response to urban floods follow a normal or abnormal resilience pattern cannot be explained by their socio-demographic attributes, indicating that a universal behavioral mechanism of disaster-avoidance responses exists across populations, regardless of the mobility disparities that associates with their gender and age.

## DISCUSSION AND CONCLUSION

We studied the resilience patterns of human mobility to an extreme pluvial flood caused by climate-triggered heavy rainfall. Drawing from a massive amount of mobile-phone-based mobility data, our findings provide new evidence to better understand the complexity of collective human movement behaviors. While data-driven and complex-system-based approaches are necessary for the studies of disaster resilience [47], it is important to acknowledge that the data used in this study suffer from several limitations. In many instances, mobile phone data cover only a portion of the entire population with selected demographic labels, and might also suffer from imbalance and representative issues in multiclass analyses. For example, data from age group 1 might not be representative as children under 10 years old normally do not possess their own smartphones in China and their mobility recordings could in fact reflect the movement patterns of their parents as they normally travel with family adults [48]. However, these are common limitations of studies that use similar data in different geographical contexts [24,30].

More than this, the mobile phone dataset that we use is on an aggregated level from a single region in China and thus cannot offer informative insights about individual behavioral trajectories. Notably, as argued by Smiley *et al.* [49], the single-event and single-region features of this type of investigation are also advantages here as we indeed provide a more in-depth scrutiny of the specific link between the resilience of collective human mobility and a specific, representative pluvial flood disaster in China. Our investigation yields several important implications.

Previous studies have already shown that human mobility highly hinges on historical behaviors and frequent visits in the past [50]. However, due to unexpected disasters and shocks, regular travel behaviors, including travel mode choices, can be significantly altered [13]. In addition, it is also worth noting that the nature of different crises can alter psychological and social conditions and thus behavioral responses of human mobility in different ways [30]. From the results, we found that intense urban floods can trigger obvious reductions in large-scale human mobility, but the overall characteristics of structural dynamics can remain stable, which implies that people’s daily mandatory trips might not be drastically changed by such acute rainfall-related floods. And this might be due to the fact that flood waters have reduced the overall mobility intensity but not fundamentally the connections at many peripheral areas in the networks. Indeed, most of the impacts were reported in the central region within the inner city, where the metro system collapsed during the event, causing massive delays and tragic death and injuries [44]. For practical responses, this finding highlights the need for maintaining the normal functionality of critical infrastructures during such emergencies to provide essential services for stable mobility dynamics and demands.

Moreover, it is also worth noting that the actual characteristics of pluvial floods at different locations could be highly heterogeneous, meaning that the actual impacts of this Zhengzhou ‘720’ flood could show greater variations at a micro-level scale in terms of impeding human mobility. For example, flood-water depths and waterfront arrival times could vary significantly in this large-scale flood event (i.e. heavy rainfall events do not hit study areas at the same time, magnitude and location). This could lead to different response times and imbalanced infrastructure damages in the built environment, which then in turn result in the heterogeneous patterns of how people react to this event at different places with different geographical contexts, and, thus, showing the diverse patterns of mobility resilience, as we observed in the analysis results. These micro-level details of flood characteristics can also offer valuable implications for decision-makers to better manage the post-disaster recovery (e.g. an informative flood map can support damage assessments to facilitate the development of priorities for a recovery agenda) and possible reconfiguration of urban spaces in future infrastructure planning (e.g. flood-proof measures could be tailored for new infrastructure facilities).

From the perspective of mobility resilience in demographic groups, we found great disparities in human mobility resilience across population groups. This means that it is certainly not feasible to find a generic, one-size-fits-all managerial solution for mitigating flood risks and improving mobility resilience for diverse populations. Regarding different gender and age groups, male and working-aged groups were more resilient than females, youth and older adults. Such disparities in mobility resilience among demographic groups can be quantitatively verified by their significantly lower probabilities of travel frequency during the event. Disparities in human mobility can affect disadvantaged groups in unexpected ways during climate-induced flooding [49]; our findings enrich the understanding of the disproportional impacts of disasters on vulnerable groups and explain their relatively lower mobility resilience and longer periods of post-disaster mobility restoration [51,52]. Additionally, it is straightforward to imply that decision-makers in local governments and response agencies play a critical role here. Local authorities have the responsibility for early warning, financial resource allocation, quick response, effective rescue and more [53]. Even though many flood disasters are initially triggered by natural hazards, the local government holds the key to preventing subsequent human errors that might lead to a nature-and-manmade mixed tragedy eventually. In practice, this means that, when developing flood-risk prevention solutions (such as sponge city programs and disaster contingency plans), policy- and decision-makers should take ‘not-happen-yet’ record-breaking future hazards into account and prioritize the implementation of disaster preparedness strategies via community and citizen engagement activities [54], including public education on disasters during normal times and more targeted and structured emergency training practices concerning the vulnerable.

In terms of mobility resilience patterns in space, to the best of our knowledge, this study is the first to uncover the existence of a significant amount of abnormal resilience patterns in spatial-temporal collective human mobility in response to large-scale extreme urban floods. The ratio between normal and abnormal patterns roughly followed 1:1 in this case study, and we speculate that this particular ratio may be associated with the proportion of risk-prone areas (i.e. places impacted by the flood) in the entire study area (see Figs S15–S16 in Section 4.1 of the online supplementary material for additional supporting evidence). Most importantly, we observed that this flood disaster could counter-intuitively stimulate more intense movements in certain places (recall the distribution of the second and third quadrants in Fig. 5). These abnormal behaviors are indicative of individuals changing their normal travel behaviors to avoid flooded areas and quickly adapt to disturbances, and this explains why those places of abnormal resilience patterns are distributed around the peripheries of places of normal resilience patterns and form narrow corridors. With that being said, it is also of substantial importance to note that how exactly individuals would alter their travel behaviors to adapt to flood shocks, including travel route and mode choices, is strongly related to the local geographical context in the built environment. For decision-makers, extra attention should be paid to those spots of abnormal resilience patterns as it could imply (1) potential micro-level changes of land use in the long run and (2) sudden surges of resource demand for quickly accommodating crowd agglomerations during emergencies.

Following this, it is interesting to observe that these abnormalities are essentially not associated with people’s demographic attributes. Since many studies have shown that travel behaviors (such as mode choices, travel distance and more) have strong associations with travelers’ socio-demographic attributes, what we discovered here could potentially be a caveat; that is, the answer to the question of whether the spatial-temporal agglomeration and diffusion phenomenon of human mobility, in response to urban floods, follow normal or abnormal resilience patterns might not be explained by travelers’ gender and age (i.e. there is a universal behavioral mechanism beneath disparities during flood emergencies). Indeed, disaster-induced behavioral changes, such as hazard-avoidance behaviors, can be inherently and predominantly influenced by the disaster’s characteristics per se and this should be a universal mechanism across populations. As such, our findings illustrate this underlying mechanism and offer a new window for looking beneath the mobility disparities in different population groups.

## METHODS

### Data

The data collected for this research include (1) mobile phone data from July 13 to 30, 2021 in Zhengzhou, China; (2) basic geographic information including DEM and administrative data from Landsat 8 digital productions in Zhengzhou; (3) meteorological recordings of hourly precipitation in Zhengzhou from July 13 to 30, 2021 and (4) social media data of rescue activities collected from Weibo with labels about heavy rainfall in Zhengzhou.

The mobile phone data were provided by a third party, and all data collection activities were monitored under a mutually agreed upon approach and followed a data protection-compliant framework. The data contain 1.32 billion high-fidelity geospatial records from 4.35 million travelers’ mobile phone devices, covering information on the collective number of travelers in each spatial unit grid on an hourly basis for each observation day, including their gender and age, and location coordinates of their travel origin and destination.

The study area was cellularized into 1 × 1 km^2^ grids for the following reasons. First, this dimension can capture most of the mobility information from the users as the data were recorded from the signaling towers of the service provider, which are roughly spaced 1-km apart. Second, this 1-km dimension is closely related to the typical design of Chinese city blocks, where commercial facilities, science and education buildings, residential areas, offices and other life services are normally locate within 15-minutes walking distance, which has long been adopted as the maximum neighborhood range.

### Calculation of mobility resilience scores and statistical tests

We used a time-series, systemic, performance-based metric to quantitatively evaluate human mobility resilience. As shown in Fig. S7 in Section 2.2 of the online supplementary material, a typical resilience curve reflects the system’s behavior and describes its continuous performance level before, during and after an external disruptive event. The shaded area refers to the profile of performance loss, and performance-based resilience can be calculated as


(1) \begin{eqnarray*} {\rm RI}=\frac{\int _{t_{0}}^{t_{2}}{\rm AP}(t)\, dt}{\int _{t_{0}}^{t_{2}}{\rm NP}(t)\, dt}, \end{eqnarray*}

where RI is the resilience score, AP(*t*) is the actual performance level and NP(*t*) is the expected performance level. Because the resilience metric we adopted has a value range of [0,1], it is intuitive and easy to conduct a cross-comparison. Furthermore, we defined the percentage change of mobility flux in this study as the *y* axis of the performance profile. The percentage change can be calculated as


(2) \begin{eqnarray*} \text{Percentage change} = \bigg (\frac{x_{i}-x_{0}}{x_{0}} \bigg )\times 100\%, \end{eqnarray*}

where *x_i_* is the hourly total flux of each day and *x*_0_ is the hourly average flux on July 16 to 18 (with the pre-event stage as the benchmark). The normalization can be performed as


(3) \begin{eqnarray*} y_{i,\! j}^{*}=\frac{y_{i,\! j}-\min (y_{\! j})}{\max (y_{\! j})-\min (y_{\! j})}; \end{eqnarray*}

see Section 2.2 within the online supplementary material for the detailed steps of data curation for calculating the resilience scores. Once the calculated resilience scores were obtained, we then applied a one-way ANOVA test to statistically verify the meaningful differences across all groups. After the overall ANOVA test, Tukey’s Honestly Significant Difference (HSD) test was also applied at a 95% confidence level to further confirm the differences in population groups in a pairwise manner. These two tests are standard statistical tests that can be deployed in packages. Here, we jointly utilized R (v4.2.2) (functions *‘aov’* and *‘TukeyHSD’*) and Microsoft Excel (v16.16.1) for all the statistical tests (see Section 2.3 within the online supplementary material for the test results).

### Penalized multinomial logistic regression model

Before modeling the four types of resilience patterns, we first checked the multicollinearity issue of the explainable variables (i.e. the independent variables of gender and age) using VIF where normal multicollinearity can be confirmed if VIF > 10. Because mapping resilience patterns onto quadrant dimensions returns categorical choices, modeling with such dependent variables is a typical discrete choice problem. We applied multinomial logistic models to depict the relationship between the response probabilities and the independent variables. The regression models were fitted using an R in-built function, *multinom*. As in other forms of linear regression, MNL regression employs a linear predictor function *f* (*k, i*) to forecast the probability that observation *i* has outcome *k* and can thus be expressed as


(4) \begin{eqnarray*} f(k,i)=\beta _{0,k}+\beta _{1,k}x_{1,i}+\cdots +\beta _{M,k}x_{M,i}, \end{eqnarray*}

where β_*m,k*_ is the coefficient associated with the *m*th explanatory variable and the *k*th outcome. Next, we applied penalty weights to the four quadrant classes to address the imbalanced class problem. This penalized model avoids data manipulation by assigning penalty weights to multiple classes. Given a series of multiclasses as 1, 2, 3, …, *n*, the penalty weight for class *i* can be determined as $w_{i} = {[(\sum _{i=1}^{n}N_{i})/n]}/{N_{i}}$, where *N_i_* is the number of observations in class *i*. In this study, the weights for Q1, Q2, Q3 and Q4 are 2.616, 0.808, 3.454 and 0.479, respectively.

We then performed a series of stepwise regression analyses to explore the individual explanatory strength of each explainable variable (i.e. gender and age). The process of the stepwise regression analyses involves starting with the basic model with the intercept only, and manually introducing the explainable variables one by one into the model, and then checking the resultant change in the model’s deviance. By doing so, one can see which explainable variables would help reduce deviance more for better model predictability, thus ensuring a better explainable strength. Finally, for model accuracy tests, we implemented the R in-built function *‘roc$\_$curve’* to conduct the receiver operating characteristic (ROC) analysis. From the corresponding confusion matrix, one can calculate the AUC values to depict each model’s overall accuracy (note that the closer the ROC curves are to the diagonal line on the plot, the worse the model’s accuracy).

### Network modeling using the DBA model

We refer the reader to Section 2.4 within the online supplementary material for full details on the model construction and simulation settings.

## AVAILABILITY OF DATA AND CODE

The mobile phone data were used for research purposes only. The authors are not allowed to share these data, in any possible form, or otherwise make public the data with any party or individual. Other datasets are publicly accessible. The analysis code supporting the findings of the present study can be freely downloaded online on GitHub at https://github.com/Fe6ruAr9/Zhengzhou_Project.

## Supplementary Material

nwad097_Supplemental_File
