# Supplementary material for: Resilience patterns of human mobility in response to extreme urban floods
Source: Natl Sci Rev. 2023 Apr 11;10(8):nwad097. doi: 10.1093/nsr/nwad097 (PMC10306362; doi:10.1093/nsr/nwad097)
Supplement: nwad097_Supplemental_File [file nwad097_supplemental_file.pdf]

Supplementary information for

Resilience patterns of human mobility in response to extreme urban floods

Junqing Tang[1,2], Pengjun Zhao[1,2,3]*, Zhaoya Gong[1,2], Hongbo Zhao[4], Fengjue Huang[1], Jiaying Li[1], Zhihe Chen[1], Ling Yu[1], and Jun Chen[5]

[1] School of Urban Planning and Design, Shenzhen Graduate School, Peking University, Shenzhen, 518055, China
[2] Key Laboratory of Earth Surface System and Human-earth Relations of Ministry of Natural Resources of China, Shenzhen Graduate School, Peking University, Shenzhen, 518055, China
[3] School of Urban and Environmental Sciences, Peking University, Beijing, 100871, China
[4] Key Research Institute of Yellow River Civilization and Sustainable Development & Collaborative Innovation Center on Yellow River Civilization, Henan Province and Ministry of Education, Henan University, Kaifeng 475001, Henan, China
[5] Key National Geomatics Center of China, Beijing 100830, China

*Corresponding author: Pengjun Zhao. E-mail: pengjun.zhao@pku.edu.cn

This PDF file includes:

Supplementary text

Figures S1 to S16

Tables S1 to S8

Supplementary references

Part I. Background information of the flood event

1.1 Study area

The study area, the Zhengzhou region (Figure S1), is located in Henan province, the north-central area of China (112°42′E–114°14′E, 34°16′N–34°58′N), and covers approximately 7,446 km^2. In 2021, the region's resident population was about 12.7 million, with an urbanization rate of 79.1%. This area has a semiarid subtropical climate, an annual mean temperature of 14.35°C, and an annual mean rainfall of 640.9 mm, with the Yellow River running through it. The region is the political, economic, technological, and educational center of the province and also the most critical transportation hub in central mainland China. Since 1992, China's central government has designated Zhengzhou as one of 11 "Hinterland Open Cities", allowing access to the same preferential policies as the eastern coastal open cities. Currently, with the promotion of the "One Belt, One Road" initiative, the Zhengzhou region has become an important node on the "New Silk Road" between Europe and Asia.

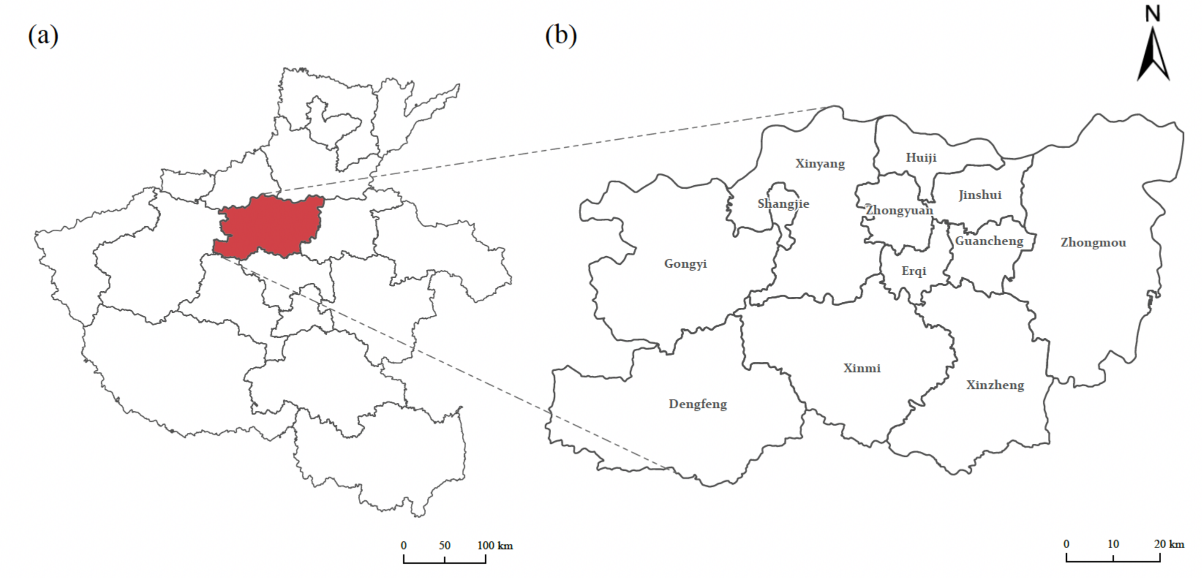

Figure S1. The study area: the Zhengzhou region in China. (a) The Henan province; (2) The Zhengzhou region.

1.2 Event description and timeline

The information on the event timeline was collected from the Disaster Investigation Team of the State Council. As depicted in Figure S2, the rainstorm center moved southward to the Zhengzhou region on July 19, 2021, which led to a long-lasting heavy rainfall event. The severe pluvial flood in Zhengzhou began on July 20, gradually lessened, and ended on July 23 (the event is soon reported with the name '720' Zhengzhou flood). From 8:00 am on July 17 to 8:00 am on July 23, the accumulated precipitation exceeded more than 600 mm and reached 5,590 km^2, and Erqi, Zhongyuan, and Jinshui districts in the central urban area had an accumulated rainfall of nearly 700 mm. The heaviest rainfall period happened between the afternoon of July 19 and the morning of July 21, with a maximum daily rainfall of 624.1

mm being recorded at Zhengzhou National Weather Station on the 20th, which is almost equal to the average annual rainfall in the Zhengzhou region (640.8 mm). This event broke the historical record of extreme meteorological observations in mainland China.

The event timeline and emergency responses are recorded in Figure S3. A quick summary of this extreme weather event is as follows: (1) This heavy rainfall caused a wide-range large-scale urban flood and was extremely intense during a relatively short period. (2) Major rivers in the region repeatedly faced grim inundation situations. (3) The inner city drainage system was unable to deal with this sudden burden. (4) Even though there were several rounds of emergency alerts before the heavy rainfall, the event still caused massive losses to society.

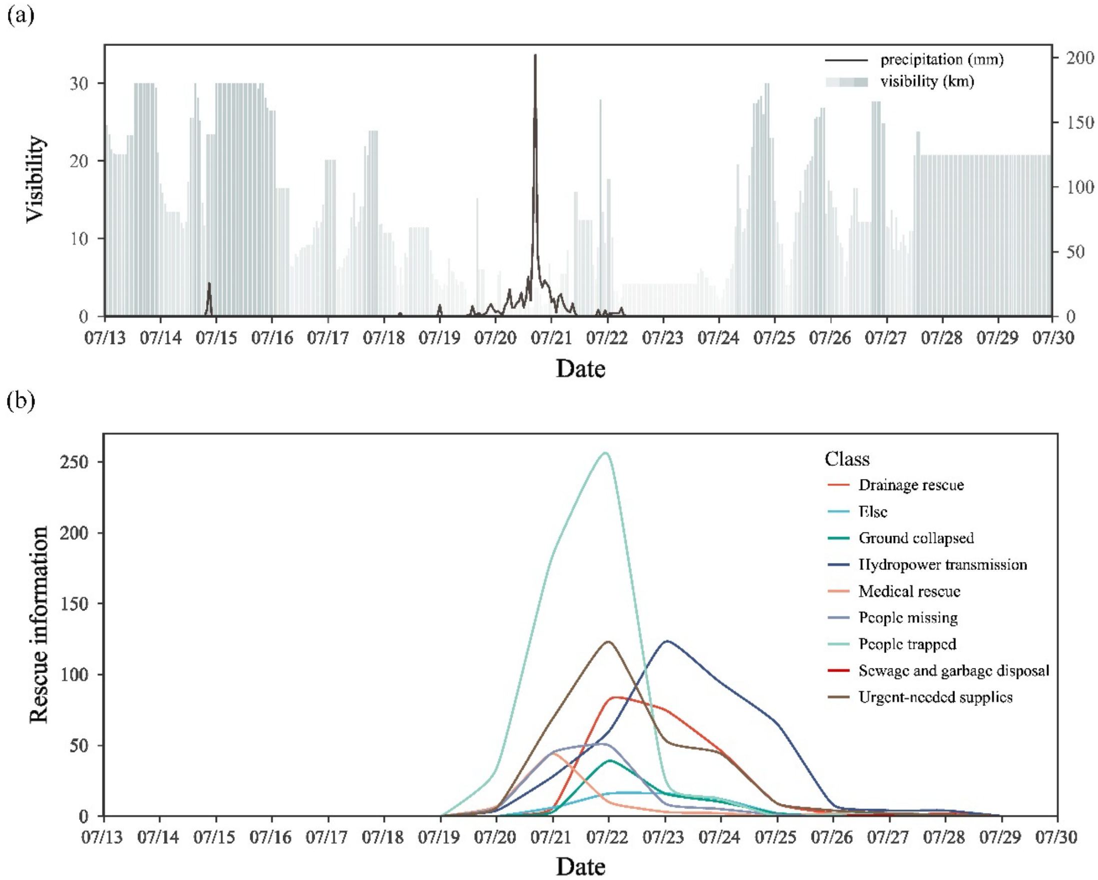

Figure S2. Time series of the rainfall process and collection data of rescue information from July 13 to 30, 2021. (a) daily weather conditions of precipitation and visibility; and (b) the rescue information was collected from social media platforms. Different colors were used to distinguish the type of search and rescue activities.

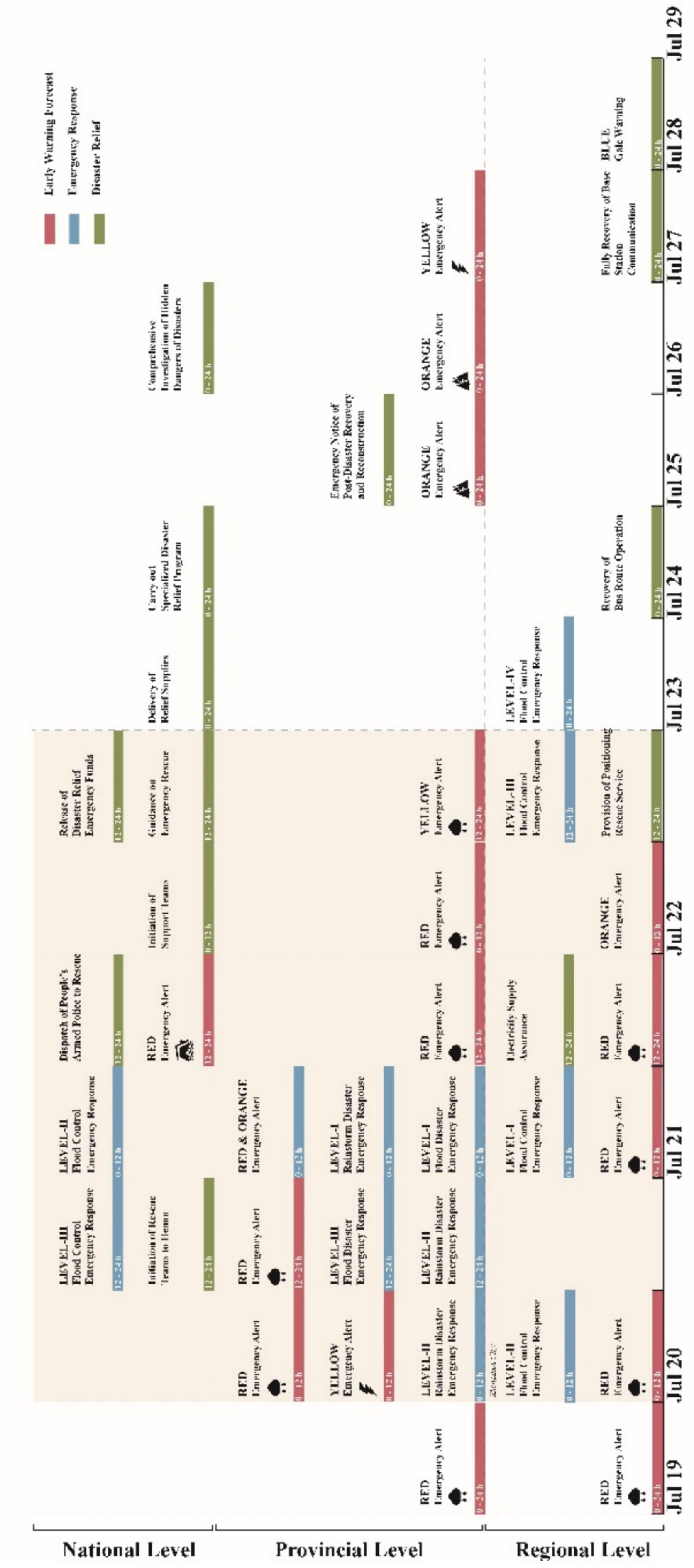

Figure S3. **Chain of events displayed at the national (top), provincial (middle), and regional (bottom) level from July 19 to 29, 2021 in the Zhengzhou region.** Red blocks represent early warning forecasts, blue blocks represent emergency response, and green blocks relate to disaster relief information.

Part II. Mobility resilience levels in demographic groups

2.1 Spatial-temporal changes in overall mobility

We discretize the study area into 1 km-by-1 km grids and subsequently use the collective human mobility OD data at pre-, during, and post-disaster stages to analyze the frequency of average daily flux. It is found that the frequency distribution of daily average flux is extremely polarized, with most of the grids occupied with fewer OD movements. We found two critical points in the distribution plot at 50 and 100 intensity levels. Thus, we reclassify the mobility data into three groups: (1) less than 50, (2) more than or equal to 50 but less than 100, and (3) more than or equal to 100 OD trips (Figure S4).

To support Figure 1 in the main manuscript, we also analyze the mobility network changes together with the network centrality measures for $G < 50$ and $50 < G < 100$ in Figures S5 and S6. Similar patterns as discussed in the main manuscript can be found at these two levels of intensity.

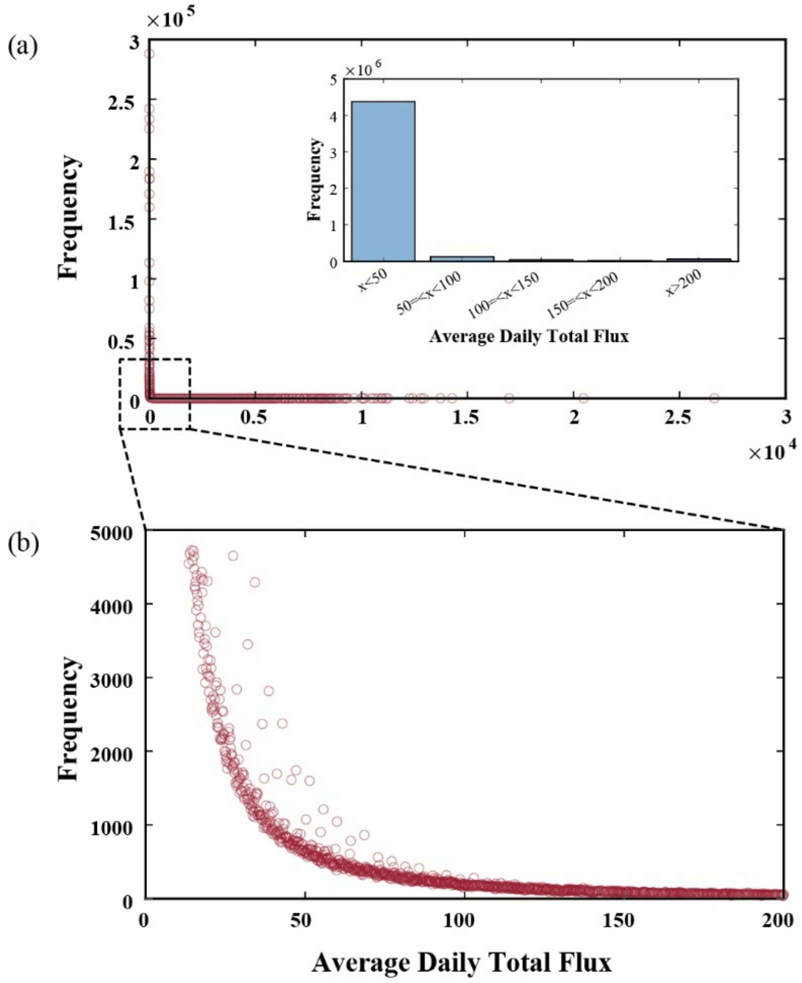

Figure S4. Frequency distribution of daily average flux.

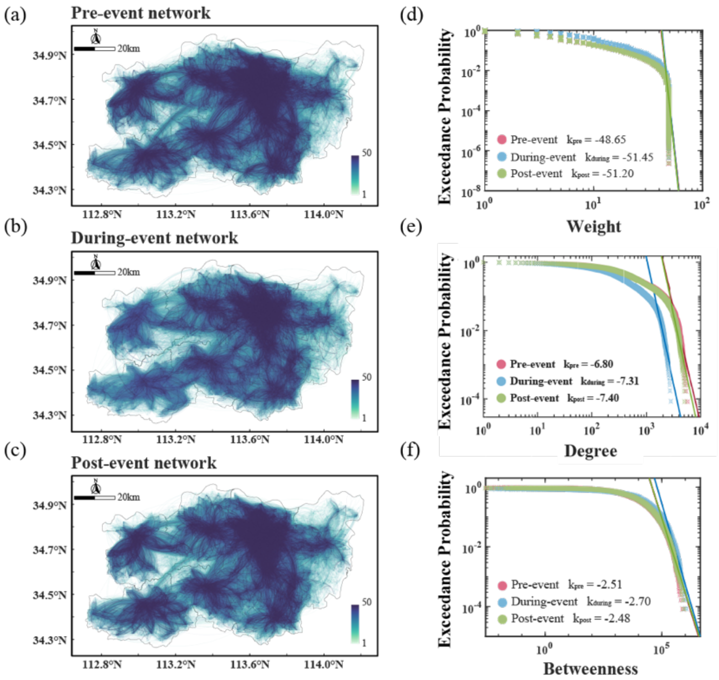

Figure S5. Same as Fig.1 in the main manuscript, but for the patterns calculated for groups that have hourly flux under 50. (a) the network flow at the pre-event stage; (b) the network flow at the during-event stage; (c) the network flow at the post-event stage; (d-f) exceedance probability with size for degree and betweenness in pre-event, during-event, and post-event stages respectively; (g-j) percentage change of degree, edge weight, betweenness, similarity from July 13 to 30. Note that the red blocks highlight the during-event process.

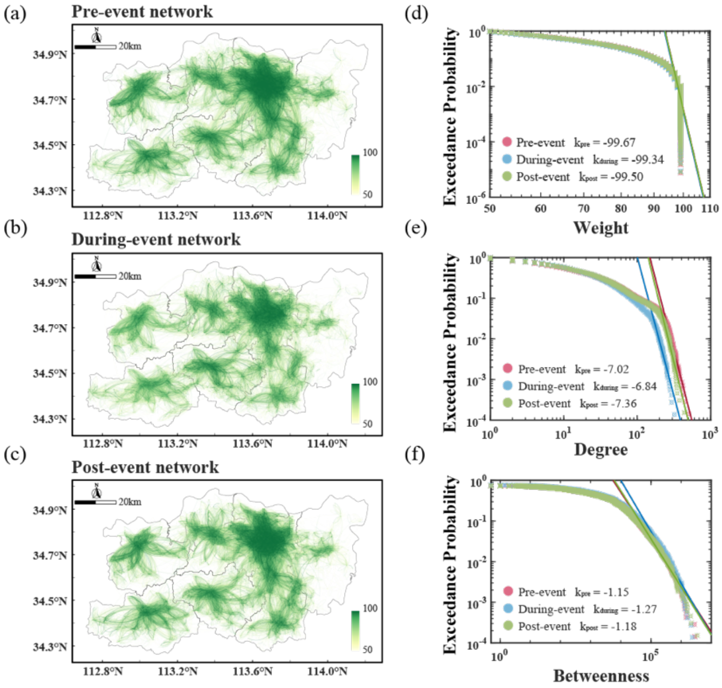

Figure S6. Network analysis for groups that have an hourly flux between 50 and 100 OD trips.

2.2 Resilience quantification metric

We use the time series systemic performance curve to quantitatively evaluate human mobility resilience (Hong et al., 2021; Kontokosta & Malik, 2018). As shown in Figure S7, a typical resilience curve reflects the system behavior and describes the continuous system performance level before, during, and after an external disruptive event (Poulin & Kane, 2021). The shaded area refers to the profile of performance loss, and the performance-based resilience can be calculated as follows (Ouyang & Duenas-Osorio, 2012):

$$RI = \frac{\int_{t0}^{t2} AP(t)dt}{\int_{to}^{t2} NP(t)dt} \tag{1}$$

where RI is the score of the human mobility resilience defined in this paper, $AP(t)$ is the actual performance level (e.g. the area under the bold black line), and $NP(t)$ is the expected performance level, which is the total area under the dotted straight line (i.e. the rectangular area). The metric proposed by Ouyang and Duenas-Osorio (2012) is widely applied for two reasons: (1) the value of resilience is calculated at a range of [0,1], which is intuitive for diagnosis and intercomparison; and (2) the metric features excellent effectiveness and simplicity for applications in empirical analysis.

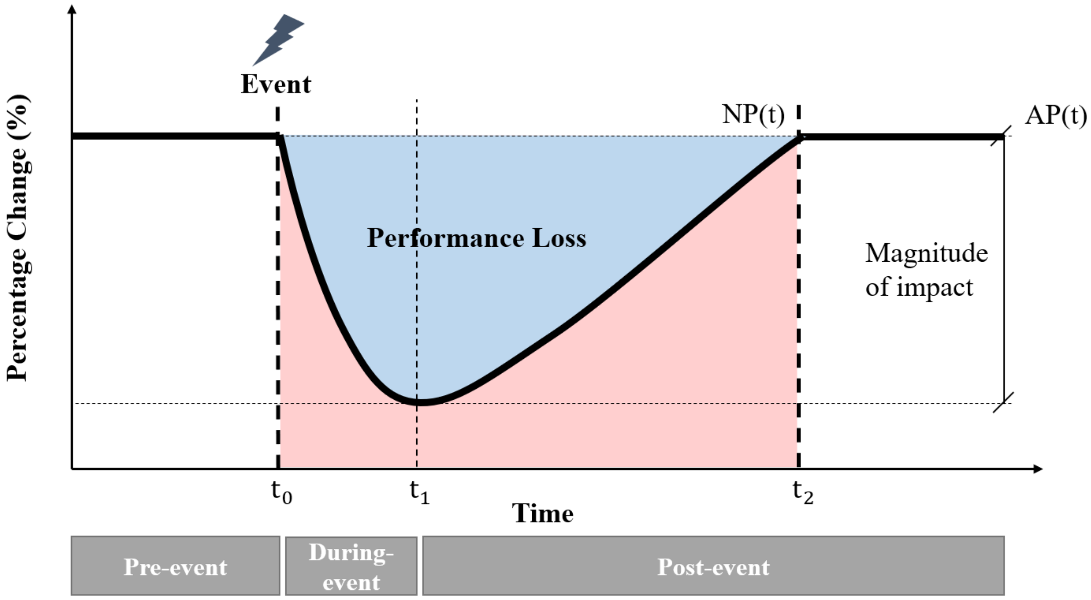

Figure S7. The conceptual resilience curve (modified based on Hong (2021) and Kontokosta and Malik (2018)). It shows the evolution of a performance measure across pre-event, during-event, and post-event stages. The maximum depth of the curve reflects the magnitude of impact.

We used the percentage change of mobility flux in this study as the y-axis of the performance profile, which features excellent effectiveness and simplicity for applications. We convert the hourly total flux to plot the resilience curves of human flow networks. Since the resilience metric we adopted has a value

range of [0,1], it is intuitive and easy to conduct cross-comparison. Thus, the percentage change can be calculated as follows:

$$Percentage\ change = \left(\frac{x_i - x_0}{x_0}\right) \times 100\% \tag{2}$$

where x_i is the hourly total flux of each day and x_0 is the hourly average flux of July 16 to 18 (with the pre-event stage as the benchmark). The normalization can be performed as:

$$\frac{y - y_{min}}{y_{max} - y_{min}} \tag{3}$$

The steps for preparing the performance profile (i.e., the resilience curve) and calculating the resilience scores are shown below.

Step 1: We calculate the total flux (i.e., the total edge weight of the network) per hour per day for each demographical group. For each group, there are 408 data points for a total of 24 hours per day for the full-length observation window of 17 days.

Step 2: Then, we set a pre-event mobility-performance benchmark to facilitate the conversion from mobility flux to percentage changes using Eq. (2). The benchmark is the averaged hourly flux from July 16 to 18 (i.e., three consecutive days before the disaster hit).

Step 3: Next, we use a simple moving average algorithm to smooth and denoise the time-series curves with a sliding window size of 24 hours.

Step 4: Finally, we apply Eq. (3) to normalize the data and Eq. (1) with the '*trapez*' function to calculate the resilience scores for all groups. All calculations were performed in R (v 4.2.2).

2.3 One-way ANOVA test on the mobility difference and resilience scores among demographical groups

We applied a one-way ANOVA test to statistically verify the differences among the percentage changes of the hourly total flux in all demographic groups between 13th July and 29th July. By doing so, we could test the statistical differences in those calculated resilience scores as well. The null hypothesis is: "The means (of the percentage changes of hourly total flux) of all the demographic groups are equal", and the alternative hypothesis is: "The means of two or more groups are significantly different from that of the others". After the ANOVA test, we then applied Tukey's HSD test at the 95% confidence level to further indicate the differences in population mean in a pairwise manner for a closer check. The test returns results on the difference in the observed means (Diff), the Lower bound and Upper bound of the confidence interval, and p-values (Table S1). R (v 4.2.2), and Microsoft Excel (v16.16.1) were jointly used here.

From the ANOVA and Tukey's HSD results, we can see that overall there are differences among demographic groups, and this difference was most statistically significant between the pair among gender groups. Eight pairs among age groups also show a statistically significant difference, while pairs

between age groups having a minor difference in resilience scores (<0.05) show a statistically insignificant difference in population means, suggesting that those age groups with similar population attributes yield a similar level of resilience.

Table S1 Results summary of one-way ANOVA test and Tukey multiple comparisons

**One-way Anova**	Df	Sum Sq	Mean Sq	F value	Pr(>F)
Demographic groups	7	0.99	0.142	14.59	<2e-16 ***
Residuals	3256	31.69	0.00973	N/A	N/A

**Tukey's HSD**	Diff.	Lower bound	Upper bound	p value	Confidence Interval
Male-Female	0.03548845	0.02174223	0.04923468	5.00E-07	95%
Age group1-Age group 2	-0.041106259	-0.06071386	-0.021498654	0.0000000	95%
Age group1-Age group 3	-0.046972211	-0.06657982	-0.027364607	0.0000000	95%
Age group1-Age group 4	-0.038800332	-0.05840794	-0.019192727	0.0000003	95%
Age group1-Age group 5	-0.039595511	-0.05920312	-0.019987907	0.0000001	95%
Age group1-Age group 6	-0.012979216	-0.03258682	0.006628388	0.4098386	95%
Age group 3-Age group 2	0.005865953	-0.01374165	0.025473557	0.9572786	95%
Age group 4-Age group 2	-0.002305927	-0.02191353	0.017301678	0.9994407	95%
Age group 5-Age group 2	-0.001510747	-0.02111835	0.018096857	0.9999301	95%
Age group 6-Age group 2	-0.028127042	-0.04773465	-0.008519438	0.000631	95%
Age group 4-Age group 3	-0.008171879	-0.02777948	0.011435725	0.8424733	95%
Age group 5-Age group 3	-0.0073767	-0.0269843	0.012230905	0.8921933	95%
Age group 6-Age group 3	-0.033992995	-0.0536006	-0.014385391	0.0000121	95%
Age group 5-Age group 4	0.00079518	-0.01881242	0.020402784	0.9999971	95%
Age group 6-Age group 4	-0.025821116	-0.04542872	-0.006213511	0.002437	95%
Age group 6-Age group 5	-0.026616295	-0.0462239	-0.007008691	0.00155	95%

Signif. codes: 0 ‘***’ 0.001 ‘**’ 0.01 ‘*’ 0.05 ‘.’ 0.1 ‘ ’ 1

1 Age group 1 - 0~18 years old
2 Age group 2 - 19~29 years old
3 Age group 3 - 30~39 years old
4 Age group 4 - 40~49 years old
5 Age group 5 - 50~59 years old
6 Age group 6 - over 60 years old

2.4 Network generation modeling: Preferential attachment model

The ability to generate random networks that can accurately represent real social contact networks is essential to the study of natural or human-made systems. The Barabási-Albert model and its extensions attempt to reflect reality by generating random scale-free networks using a preferential attachment mechanism. Here, we choose the dual-Barabási-Albert (DBA) model (Moshiri, 2018) rather than Barabási-Albert (BA) model because the latter allows only two inputs, the total number of nodes and the number of edges, to attach from a new node to existing nodes. Therefore, the BA model can only describe the strength of the network generation process and cannot be applied to measure generation uncertainties of the network's growth under different growth rates (i.e., fast growth or slow growth). The DBA model is better suited to observe such uncertainties with two controled parameters (i.e., $m1$ and $m2$).

2.4.1 Model construction

We simulate the growth and shrinkage processes of the mobility networks using the DBA model, a preferential attachment-based network generation model, to capture the evolutionary processes of the mobility networks at pre-, during-, and post-event stages. The parameters of the model encapsulated in the Python package '*Networkx*' are set as follows:

$$dual\_barabasi\_albert\_graph\ (n, m1, m2, p, seed = None, initial\_graph = None) \tag{4}$$

where n is the number of nodes, $m1$ is the number of edges to link the new node to existing nodes with a probability of p, $m2$ is the number of edges to link each new node to existing nodes with a probability of $(1-p)$[1] (Figure S8).

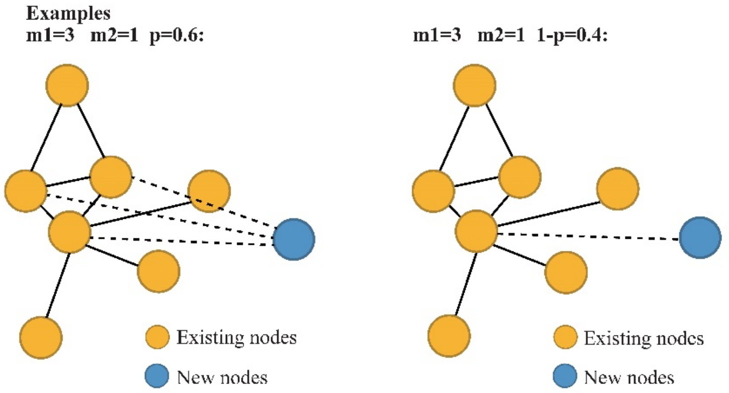

Figure S8. Schematic diagram of the generation process of the DBA network ($m1$=3, $m2$=1, p=0.6)

[1] Dual-Barabási-Albert graph: https://networkx.org/documentation/stable/reference/generated/networkx.generators.random_graphs.dual_barabasi_albert_graph.html

We focus on the parameter p in the model, which can be used to describe uncertainties in the network growth process with a faster rate (i.e., how likely the network would grow at a faster rate with adding $m1$ new edges each time step) and thus, it indicates the level of willingness to generate more travel trips. For the value range of p, we confined it between 0 and 1 with a step length of 0.02 to maximize its variations in consecutive time steps (cross-referencing Figure 3 (d-e) in the main text). By doing so, $m1$ can be set by finding the situation where the prerequisites regarding p's value range and the goodness of the power-law fittings between ground-truth networks and DBA-generated networks can be fulfilled. In addition, considering the number of edges in DBA-generated networks is always greater than the number of nodes, we set n to 1,000 (4,000 in the female and male subgroups) so that the parameter p can optimally capture the changes of the edge number in ground-truth networks and also meet the requirement on the [0,1] value range.

On the contrary, the parameter $m2$ was set as a constant (i.e. $m2$ =1), which characterizes the generation of non-multi-edge networks. The reason for such a setting is that the magnitude of $m2$ is not the main concern here as long as it can effectively represent a slow-enough growth rate. $m2 = 1$ represents a situation where only one new edge is generated in the network at each time step (i.e., as a strong contrast to a much larger $m1$ that represents a much faster growth rate). Moreover, by controling $m2$ we are able to lay a benchmark for effective comparisons in later analyses, otherwise, we would not be able to compare the performance of p based on bivariate $(m1, m2)$ pairs. The specific parameter settings of the DBA models for different demographical groups are shown in the following Table S2.

Table S2. Parameters for the DBA network generation model

group	n	$m1$	$m2$
Age group 1	1000	10	1
Age group 2	1000	215	1
Age group 3	1000	415	1
Age group 4	1000	175	1
Age group 5	1000	115	1
Age group 6	1000	45	1
Female	4000	55	1
Male	4000	100	1

2.4.2 The fitting process of the parameter p

For each demographic group, we perform 136 (files with average OD data in 3h) times 50 (values of p between 0 and 1) times 50 (Monte Carlo simulations) = 340,000 simulation runs, which would cost 14 hours for a single-core CPU with full loading under normal conditions.

The specific fitting operation for parameter p is as follows.

Step 1 - Data preparation: set the time window of the observed data to 3h and calculate the average number of trips. Re-organize the data columns into 'Sources' (i.e. the origin), 'Destinations' (i.e. the destination), and 'Weights' (i.e. the intensity of an OD connection).

Step 2 - Network modeling: construct a network model of the empirical data using '*Networkx*' Python package and count the number of edges in the network.

Step 3 - Fitting: based on the parameters in Table S2, obtain the optimal parameter p having the minimum discrepancy between the DBA-generated network and the ground-truth network by iterating through the range of p.

Step 4 - Monte Carlo simulations: perform 50 iterations and obtained the converged values as the final p outcomes.

Part III. Heterogeneous resilience patterns in space

3.1 Definition of the quadrant plot and the outliers in drawdown and drawup processes

The resilience curve uses each day minus the previous day and calculates a change ratio, generating a curve showing the change in the flow of people each day, and finally using the resilience triangle method to measure its resilience. Furthermore, for analyzing the resilience patterns (e.g. bathtub curves) in this section, we first calculate the daily average mobility flow (outflow/inflow) in each 1 km-by-1 km grid for all pre-event, during-event, and post-event stages. Next, we generate a four-quadrant distribution map, in which the x-axis is the mobility flow that averages the pre-event level minus the during-event level (i.e. the drawdown process) and the y-axis is the value of the averaged during-event level minus the level at the post-event stage (i.e. the drawup recovery process). The specific four-quadrant plotting methods are as follows: (1) we calculate the average daily human flow at the pre-, during-, and post-event stages by sparse matrix using MATLAB; (2) we calculate the outflow and inflow grouped by origin location and destination location respectively; and (3) we generate the flow difference value for the x-axis and y-axis and transform the continuous value into a Cartesian plotting.

3.2 Sub-network community analysis with respect to diverse resilience patterns

3.2.1 Community detection algorithm for identifying sub-network clusters

Here, we test how mobility patterns might change at a smaller spatial scale (the sub-cluster level of the mobility network) to see if the ratio between normal and abnormal resilience patterns could be scale-invariant. To do so, we first apply the modularity algorithm to detect the communities (i.e. the sub-network clusters) in the mobility networks under normal conditions (i.e. the pre-event condition) and classify the spatial grids into different clusters.

We apply the modularity algorithm for detection, which is often used in optimization methods for detecting community structure in networks. It can be expressed as the following algorithm (Newman, 2006):

$$Q = \frac{1}{2m}\sum_{vw}\left[A_{vw} - \frac{k_v k_w}{2m}\right]\frac{s_v s_w + 1}{2} \qquad (5)$$

As a result, seven clusters of the pre-disaster aggregated network are detected by applying Gephi with the modularity algorithm at a resolution = 2. We found a significant spatial coupling relationship between the administrative boundaries and the structure of the clusters, which indicates that most of the human mobility activities are confined by administrative boundaries (i.e. most movement occurs within the clusters, that is, within the administrative boundaries), while the movement across cluster divisions accounts for only a small number but with relatively stable intensity and frequency.

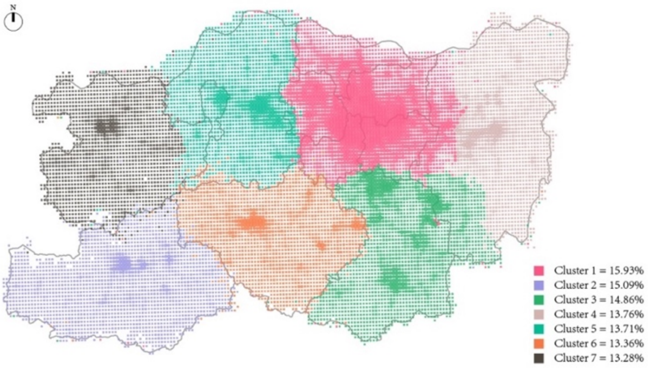

Figure S9. Community detection of the pre-disaster aggregated network. Seven main clusters are detected and labeled using different colors.

3.2.2 Significance of differences in clusters using the K-S test

We then test whether the differences among clusters are statistically significant. Table S3 presents a comparative description of all clusters in terms of the outflows in the drawdown (i.e. the pre-event level minus the during-event level) process, and it can be seen that Cluster 1 (i.e.

the one in the central urban area) has the highest amount of outflow during the disaster event and the least resilience score in the whole process, which verifies that the central urban area was severely hit and damaged. Clusters 2, 3, and 6 have a relatively higher resilience level, with Cluster 6 being the highest of all (1,611 grids; 13.36% of the total grids; mean = 10.665, with RI = 0.75). This result is in line with the observations from the spatial autocorrelation analysis suggesting that these clusters have relatively sparse disaster site distributions.

Table S3. The outflow mobility at the "before minus during" process

Cluster	Basic cluster attribute		Descriptive							Tests of Normality (Kolmogorov-Smirnov*a)		
**No.**	**Size (Grid Count)**	**Proportion**	**Count**		**Total Flux**		**Mean**	**Standard Deviation**	**Resilience Index**	**Statistic**	**df**	**Sig.**
			**Red**	**Blue**	**Red*b**	**Blue*c**						
**Cluster 1**	1921	15.93%	597	1320	-630947.143	14760942.286	7370.889	16809.645	0.42	.287	1917	.000
**Cluster 2**	1820	15.09%	809	1010	-564017.571	1112558.905	301.562	3433.894	0.73	.327	1819	.000
**Cluster 3**	1792	14.86%	577	1212	-278833.429	2139927.667	1040.299	3868.733	0.71	.307	1789	.000
**Cluster 4**	1660	13.76%	593	1063	-69023.524	546386.333	288.263	1241.103	0.68	.343	1656	.000
**Cluster 5**	1654	13.71%	636	1018	-462365.429	1554044.857	660.024	3207.302	0.60	.287	1654	.000
**Cluster 6**	1611	13.36%	778	833	-1039702.286	1056884.333	10.665	2830.725	0.75	.212	1611	.000
**Cluster 7**	1602	13.28%	550	1050	-352508.905	1479991.905	704.677	3784.321	0.50	.306	1600	.000

Lilliefors Significance Correction
Before minus during is negative, which indicates the flux of during is increasing.
Before minus during is positive, which indicates the flux of during is decreasing.

For the pairwise comparisons of clusters, we apply the Kruskal-Wallis H test (one-way ANOVA on ranks), a non-parametric method to verify the difference in the outflow distributions across all seven clusters (Kruskal & Wallis, 1952; Corder & Foreman, 2009), for those clusters that do not pass the normality test.

The result indicates that, overall, there exists a statistically significant heterogeneity of outflow distributions in clusters. The same conclusion is also obtained in the tests for outflow in the drawup process and for inflow in both the drawdown and drawup processes. The significance tests on pairwise differences show that there exist similarities between certain cluster pairs (i.e. Cluster 6 and Cluster 2; Cluster 4 and Cluster 5; Cluster 5 and Cluster 7; and Cluster 7 and Cluster 3 – the adjusted p-values are 1.00, 1.00, 0.829, and 0.157 respectively). We conclude that, even though the differences in flow distributions among clusters are significant from a holistic viewpoint, there could also be similar mobility patterns at a smaller spatial scale.

Table S4. Pairwise comparisons of clusters

Cluster Sample A - Cluster Sample B	Test Statistic	Std. Error	Std. Test Statistic	Sig.	Adj. Sig. *a
6-2	13.592	118.974	.114	.909	1.000
6-4	587.328	121.693	4.826	.000	**.000**
6-5	808.577	121.730	6.642	.000	**.000**
6-7	-1059.669	122.739	-8.634	.000	**.000**
6-3	1379.719	119.442	11.551	.000	**.000**
6-1	2511.887	117.537	21.371	.000	**.000**
2-4	-573.735	118.114	-4.857	.000	**.000**

2-5	-794.985	118.151	-6.729	.000	**.000**
2-7	-1046.076	119.191	-8.776	.000	**.000**
2-3	-1366.126	115.793	-11.798	.000	**.000**
2-1	2498.295	113.827	21.948	.000	**.000**
4-5	-221.250	120.889	-1.830	.067	1.000
4-7	-472.341	121.905	-3.875	.000	**.002**
4-3	792.391	118.585	6.682	.000	**.000**
4-1	1924.559	116.666	16.496	.000	**.000**
5-7	-251.091	121.941	-2.059	.039	.829
5-3	571.142	118.622	4.815	.000	**.000**
5-1	1703.310	116.704	14.595	.000	**.000**
7-3	320.050	119.658	2.675	.007	.157
7-1	1452.218	117.757	12.332	.000	**.000**
3-1	1132.168	114.316	9.904	.000	**.000**

Each row tests the null hypothesis that the Cluster Sample A and Cluster Sample B distributions are the same.

Asymptotic significances (2-sided tests) are displayed. The significance level is .05.

a. Significance values have been adjusted by the Bonferroni correction for multiple tests.

3.2.3 Mobility resilience in clusters

In terms of mobility resilience in clusters, Figure S10 and Figure S11 suggest that the differences in resilience between outflow and inflow are rather minor, which indicates a highly similar mobility loss in terms of people's arrivals and departures within a cluster during the whole observation window. Furthermore, it can be argued that none of the seven clusters fully recovered to the pre-event level by the end of the observation period and yielded an average inflow resilience score of 0.631 at the cluster level. Compared with the resilience scores obtained from the outflow analysis, the average outflow resilience of mobility in a cluster is 0.627.

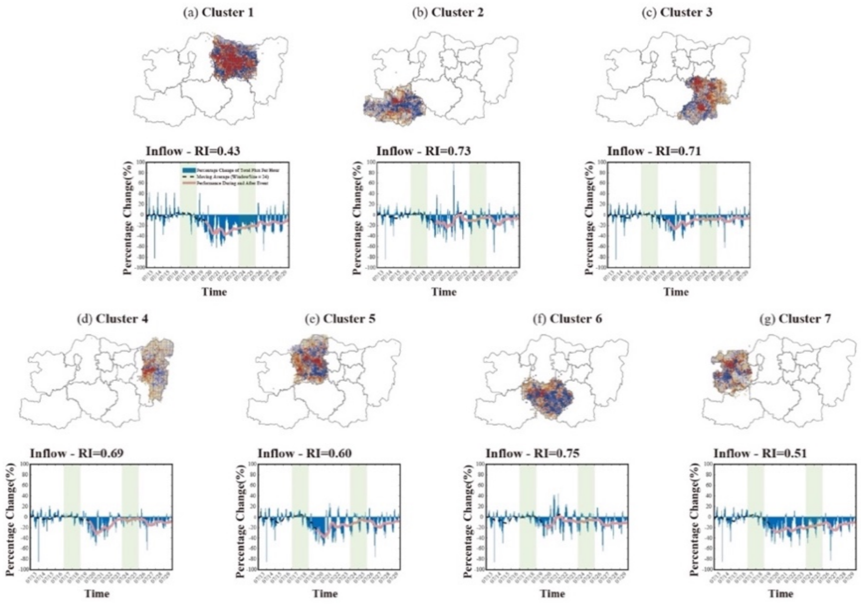

Figure S10. Mobility resilience of inflow in seven clusters.

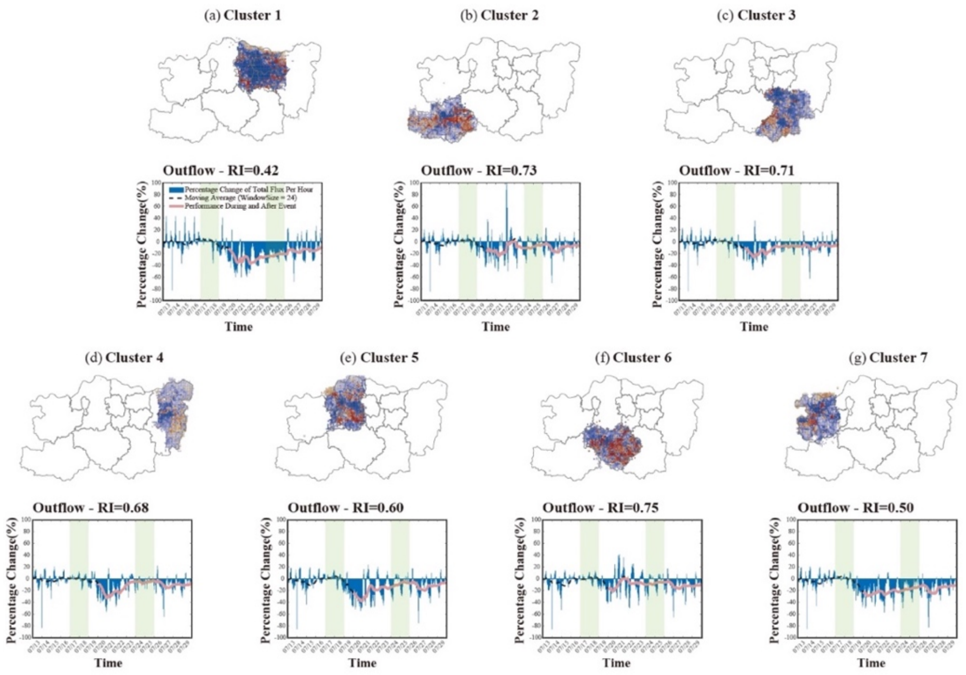

Figure S11. Mobility resilience of outflow in seven clusters.

3.2.5 Diverse resilience patterns in clusters

Figure S12 and Figure S13 show that the proportion of the four quadrants in each cluster is mostly close to the ratio of 5:3:1:1 found at the global region scale, which verifies that the ratio of normal and abnormal resilience patterns could remain stable at a smaller spatial scale.

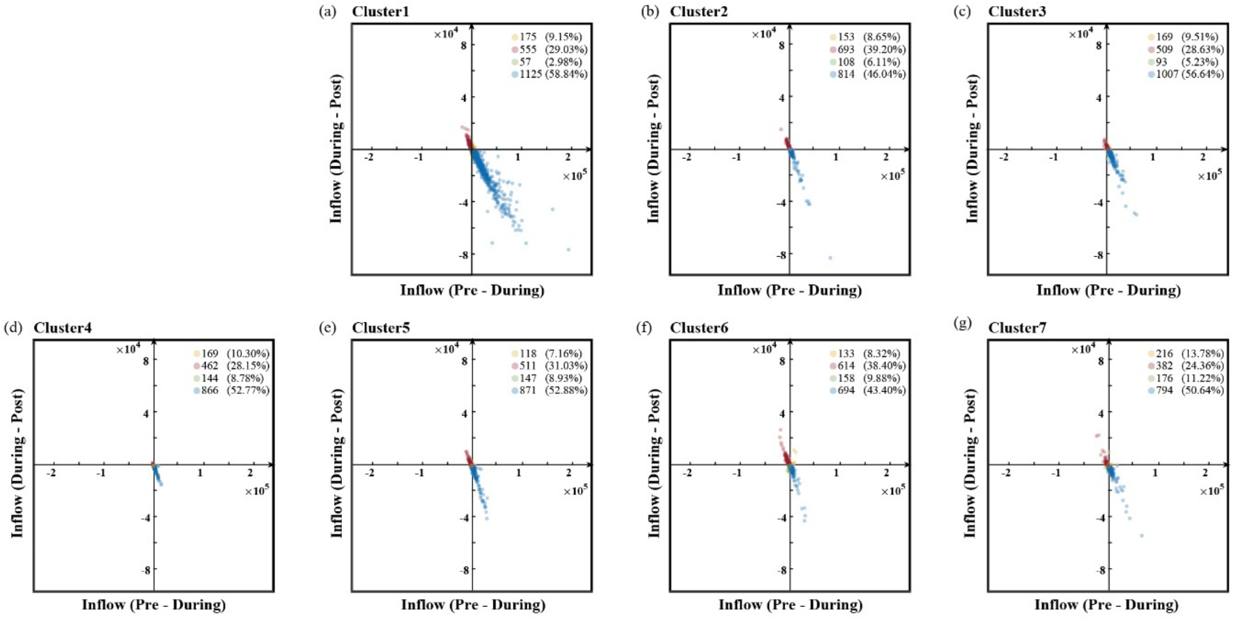

Figure S12. Counter-intuitive reversed bathtub of inflow in every cluster

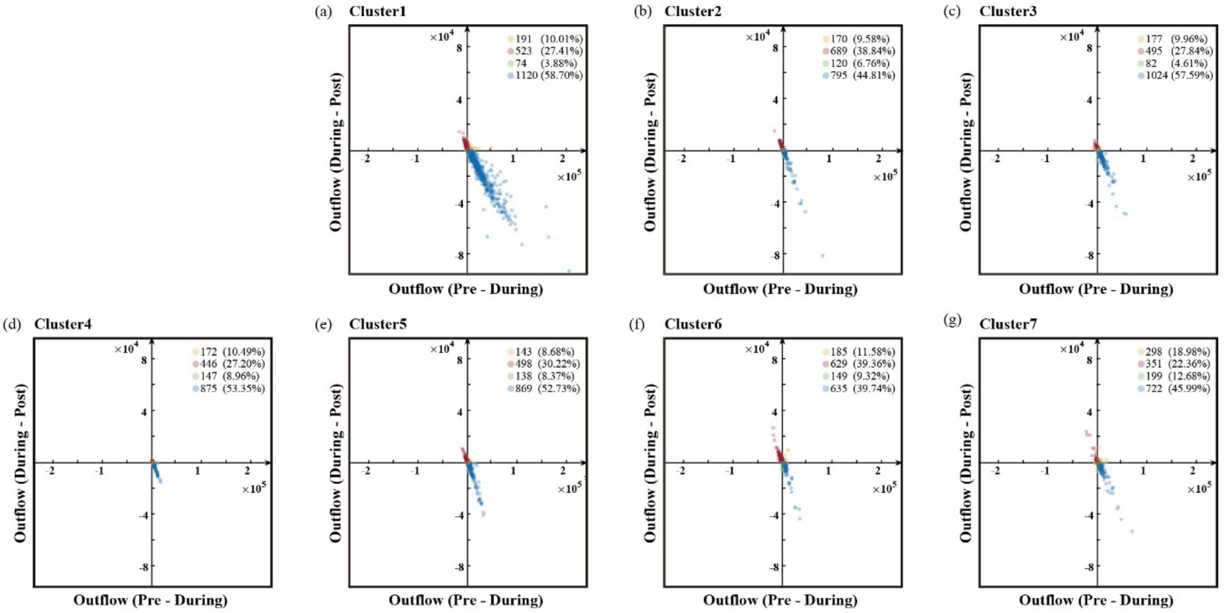

Figure S13. Counter-intuitive reversed bathtub of outflow in every cluster

3.3 Step-wise regression results and statistical tests

We adopt step-wise multinomial logistics regression to further examine the explanatory strength of demographic attributes in relation to the multi-class of quadrants. Note that, of the total records in each attribute mobility dataset (i.e. gender or age), 75% are used in the training process, while the remaining 25% are used in the validation step for calculating models' accuracy and AUC.

The step-wise regression results for the gender group are shown in Table S4. The first model (Model 0) serves as a reference and we first detect the multicollinearity between predictors by using the variance inflation factor (VIF). The high VIFs (11,288.1 for females and 7,204.0 for males) indicate a potentially strong correlation between the variables and thus they do not provide unique or reliable information in the regression Model 0. Hence, we perform two step-wise single index models (Model 1 and Model 2). As shown in Table S5, females and males do not contribute much to the reduction of residual deviance and AIC. The model of the female group has lower accuracy (0.239), while the model of the male group performs at a higher accuracy of 0.522. Each gender model's standard error and AUC are shown in Table S6. The average AUC of Model 1 is 0.578, that of Model 2 is 0.584, and the average AUC is 0.578. The results demonstrate that both female and male attributes are not necessarily associated with mobility resilience patterns.

Similar findings are also found regarding age groups. The step-wise regression results for the age group are shown in Table S7, and the models' standard error and AUC are shown in

Table S8. The VIF values of Model 0 are much higher than 5, which indicates that all the age variables are highly correlated with other variables. In terms of accuracy, all the models have poor prediction performance in the test dataset, and all the accuracy values are lower than 0.5. Model 4 (age 40–49) has the lowest residual deviance, while Model 6 (age 60) shows the highest. As shown in Table S8, all age groups have significantly small AUC values in the four quadrants, and all the average AUCs are lower than 0.6. The findings confirm that the multi-class quadrants cannot be explained by age.

Table S5 Models summary for using gender variables

**Model**	**Gender variables**	**Residual Deviance**	**AIC**	**Accuracy**	**VIF**
0	**Quadrant ~ .**	**23,606.4**	**23,624.4**	**0.260**	**-**
1	Quadrant ~ female	23,641.9	23,653.9	0.239	11,288.1
2	Quadrant ~ male	23,704.4	23,716.4	0.522	7,204.0
avg	-	23,650.9	23,664.9	0.341	9,246.0

Table S6 Models' standard error and AUC (gender variables)

**0**	**Intercept**	**Female**	**Male**	**AUC**
Q1	0.0201	5.20E-05	4.04E-05	0.512
Q2	0.0200	5.85E-05	4.49E-05	0.561
Q3	0.0200	4.82E-05	4.01E-05	0.615
Q4	-	-	-	0.605
			avg AUC	0.573
**1**	**Intercept**	**Female**		**AUC**
Q1	0.0339	8.83E-06		0.55
Q2	0.0342	1.04E-05		0.562
Q3	0.0343	1.08E-05		0.607
Q4	-	-		0.599
			avg AUC	0.578
**2**	**Intercept**	**Male**		**AUC**
Q1	0.0199	5.88E-06		0.556
Q2	0.0199	6.70E-06		0.563
Q3	0.0199	7.20E-06		0.613
Q4	-	-		0.603
			avg AUC	0.584
			avg AUC	0.578

Table S7 Models summary for using age variables

Model	Age variables	Residual Deviance	AIC	Accuracy	VIF
0	Quadrant ~ .	23,461.9	23,503.9	0.270	-
1	Quadrant ~ age0_18	23,739.7	23,751.7	0.412	5,673,875.2
2	Quadrant ~ age19_29	23,689.8	23,701.8	0.409	248.4
3	Quadrant ~ age30_39	23,608.5	23,620.5	0.235	289.7
4	Quadrant ~ age40_49	23,597.3	23,609.3	0.242	34,508.7
5	Quadrant ~ age50_59	23,654.3	23,666.3	0.278	251,892.2
6	Quadrant ~ age60	23,768.8	23,780.8	0.278	36,103.5
avg	-	23,645.8	23,662.0	0.303	999,486.3

Table S8 Models' standard error and AUC (age variables)

0	Intercept	age0_18	age19_29	age30_39	age40_49	age50_59	age60	AUC
Q1	0.0207	5.57E-04	5.01E-05	6.49E-05	0.000137	0.000193	0.000299	0.548
Q2	0.0206	6.97E-04	6.46E-05	8.08E-05	0.000160	0.000200	0.000283	0.595
Q3	0.0205	6.10E-04	6.24E-05	8.37E-05	0.000172	0.000212	0.000294	0.592
Q4	-	-	-	-	-	-	-	0.613
							avg AUC	0.587

1	Intercept	age0_18	AUC	4	Intercept	age40_49	AUC
Q1	0.0337	0.000261	0.541	Q1	0.0348	0.0000205	0.559
Q2	0.0341	0.000320	0.556	Q2	0.0350	0.0000220	0.561
Q3	0.0340	0.000316	0.608	Q3	0.0353	0.0000252	0.608
Q4	-	-	0.591	Q4	-	-	0.602
		avg AUC	0.574			avg AUC	0.583

2	Intercept	age19_29	AUC	5	Intercept	age50_59	AUC
Q1	0.0331	1.32E-05	0.514	Q1	0.0357	0.0000318	0.555
Q2	0.0336	1.88E-05	0.561	Q2	0.0356	0.0000308	0.562
Q3	0.0336	1.82E-05	0.613	Q3	0.0359	0.0000346	0.605
Q4	-	-	0.601	Q4	-	-	0.6
		avg AUC	0.572			avg AUC	0.581

3	Intercept	age30_39	AUC	6	Intercept	age60	AUC
Q1	0.0341	9.97E-06	0.55	Q1	0.0358	0.0000916	0.551
Q2	0.0344	1.20E-05	0.562	Q2	0.0355	0.0000830	0.557
Q3	0.0346	1.28E-05	0.614	Q3	0.0357	0.0000895	0.599
Q4	-	-	0.601	Q4	-	-	0.593
		avg AUC	0.582			avg AUC	0.575
						avg AUC	0.579

3.4 Normal and abnormal resilience patterns in the male group

Comparing the histograms of women and men, (1) a similar pattern is found when the majority of people are distributed in the fourth quadrant, and (2) there are slightly more men than women in each quadrant. Comparing the histogram of the elderly and the genders, (1) the proportions of the elderly in Q1, Q2, and Q3 are slightly higher than that of the two genders, and (2) the distribution of the elderly in the abnormal quadrant is mostly found on the periphery of Zhengzhou region when compared with male or female. In summary, these findings support the discussion in the main manuscript that the demographic attributes of the travelers are not strongly associated with the distribution of heterogeneous resilience patterns.

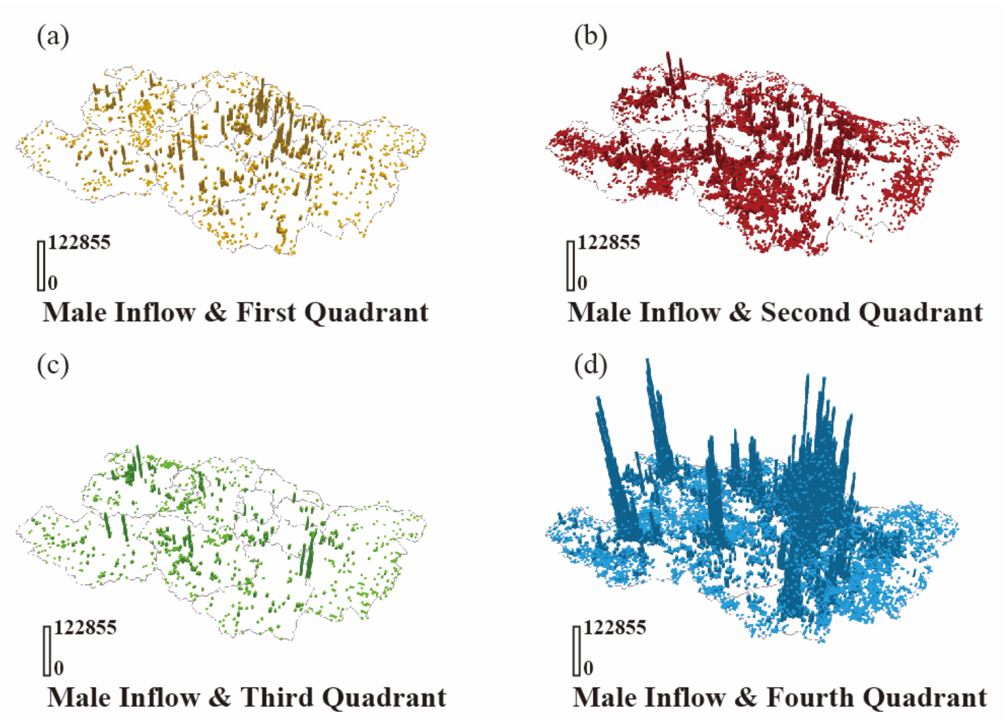

Figure S14. Male inflow visualization in the four quadrants. Note it is a 17-day daily average inflow throughout the entire event.

Part IV. Additional information for the discussion section

4.1 Spatial autocorrelation analysis

We further collect rescue data and conduct a Bivariate Local Moran's I test, a measure of spatial autocorrelation analysis to investigate how mobility changes are associated with specific damage hits (Moran, 1950; Li et al., 2007). The Local Moran's I can be defined as the following algorithm:

$$I_i = \frac{x_i - \bar{x}}{m_2} \sum_{j=1}^{N} w_{ij}(x_j - \bar{x}) \tag{6}$$

where I_i is the Local Moran's I, and N is the number of analysis units on the map.

Here, we use the location information of rescue calls as the sites that suffered from severe disaster damage, because rescue data were mostly from travelers who had been trapped in the flood or witnessed someone being severely impacted by the flood nearby and were then reported as rescue-seeking calls, which is a rational and intuitive assumption for the subsequent analysis. By setting 1km buffer zones at all rescue points, we find that, holistically, there exists a mild positive correlation (Moran's I = 0.325) between the distribution of the OD flux changes and the distribution of the disaster sites in Figure S15. Most disaster sites spatially overlap with those blue grids (decreasing outflows and calmer mobility activities) in the central urban area. By cross-referencing Figure 7 and Figure S14, we can infer that grids with a normal resilience pattern might also be correlated with the disaster sites, which further indicates that the particular ratio between the normal and abnormal resilience patterns might be associated with a portion of the disaster-impacted areas in the whole study area.

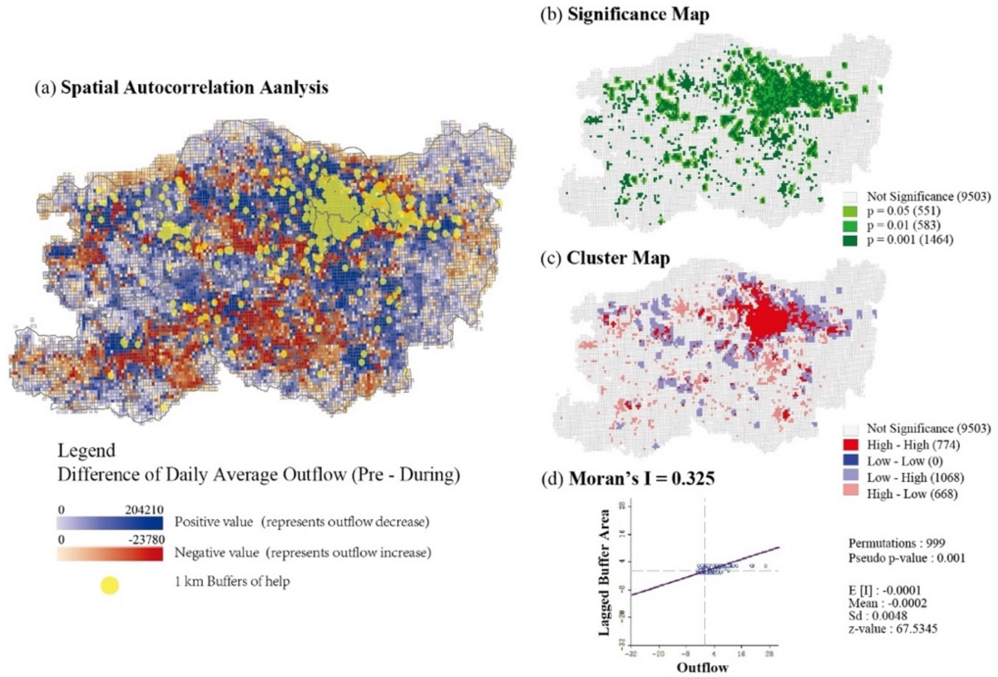

Figure S15. Spatial autocorrelation analysis between the average outflow changes in grids (the pre-event level minus the during-event level) and the disaster buffer zones. The red color indicates the rise of the average outflow while the blue color denotes the drop.

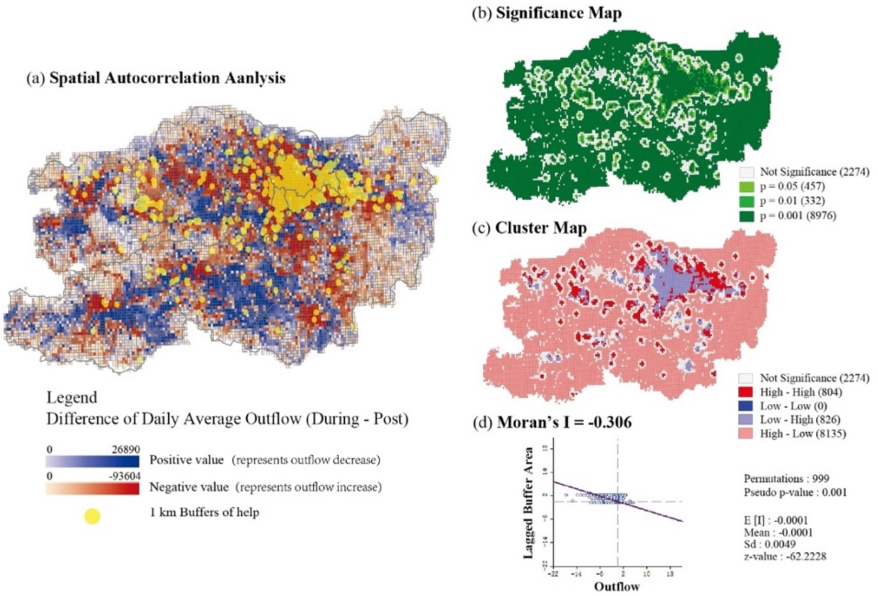

Figure S16. Spatial autocorrelation analysis between the average outflow changes in grids (the during-event level minus the post-event level) and the disaster buffer zones.

References

1. Corder, Gregory W. & Foreman, Dale I. (2009). Nonparametric Statistics for Non-Statisticians. Hoboken: John Wiley & Sons. pp. 99–105. ISBN 9780470454619.
2. Hong, B., Bonczak, B. J., Gupta, A. & Kontokosta, C. E. (2021). Measuring inequality in community resilience to natural disasters using large-scale mobility data. *Nature Communications*, *12*(1), 1870.
3. Kontokosta, C. E. & Malik, A. (2018). The Resilience to Emergencies and Disasters Index: Applying big data to benchmark and validate neighborhood resilience capacity. *Sustainable Cities and Society*, *36*, 272–285.
4. Kruskal, W., & Wallis, W. (1952). Use of ranks in one-criterion variance analysis. *Journal of the American Statistical Association*, *47*(260), 583–621.
5. Li, H., Calder, C. A. & Cressie, N. (2007). Beyond Moran's I: Testing for spatial dependence based on the Spatial Autoregressive Model. *Geographical Analysis*, *39*(4), 357–375.
6. Marsaglia, G., Tsang, W. W. & Wang, J. (2003). Evaluating Kolmogorov's Distribution. *Journal of Statistical Software*, *8*(18), 1–4.
7. Moran, P. A. P. (1950). Notes on continuous stochastic phenomena. *Biometrika*, *37*(1), 17–23.
8. Moshiri, N. (2018). The dual-Barabási -Albert model. *arXiv preprint arXiv:1810.10538.*
9. Newman, M. E. (2006). Modularity and community structure in networks. *Proceedings of the National Academy of Sciences*, *103*(23), 8577–8582.
10. Ouyang, M. & Duenas-Osorio, L. (2012). Time-dependent resilience assessment and improvement of urban infrastructure systems. *Chaos: An Interdisciplinary Journal of Nonlinear Science*, *22*(3), 033122.
11. Poulin, C. & Kane, M. (2021). Infrastructure resilience curves: Performance measures and summary metrics. *Reliability Engineering & System Safety*, *216*, 107926.
